# 2025 survey study on psychological education status among secondary vocational school students in xx province insights into mental health status and educational implications based on 31,000 students

**DOI:** 10.3389/fpsyg.2026.1773198

**Published:** 2026-08-17

**Authors:** Xiufeng Zhang, Shitao Wang, Xintong Lin, Jiayi Zheng, Lisi Li

**Affiliations:** 1 Department of Marxism, Guangdong Polytechnic Normal University, Guangzhou, Guangdong, China; 2 Department of Mathematics, Sun Yat-sen University, Guangzhou, Guangdong, China; 3 Department of Pharmacy, Guangdong Lingnan Polytechnic, Guangzhou, Guangdong, China; 4 Guangzhou Pinhui Education Technology Co., Ltd., Guangzhou, Guangdong, China

**Keywords:** mental health, psychological education, secondary vocational education, student development, survey

## Abstract

This study analyzes mental health literacy, psychological risk levels, lifestyle habits, social relationships, and academic motivation among secondary vocational students. Data reveals: Overall mental health remains stable, yet only 4.3% meet mental health knowledge standards. Academic burnout is widespread, with students struggling to derive sufficient value from academic achievements. The detection rate of depressive symptoms reached 26.35%, comparable to rates of 28% among high school students and 24% among junior high school students. Students with severe depressive symptoms also exhibit potential psychological crises such as self-harm and suicidal tendencies. The findings suggest that enhancing mental health literacy, optimizing the psychological growth ecosystem for students, empowering teacher development, and strengthening the construction of psychological crisis prevention and control networks to provide psychological support for the high-quality development of secondary vocational education.

## Introduction

To implement the “Special Action Plan for Comprehensively Strengthening and Improving Student Mental Health Work in the New Era (2023–2025)” and the mental health education initiatives of the XX Provincial Department of Education, the XX Provincial Vocational Education Moral Education Research and Guidance Center conducted a specialized survey on student mental health status in secondary vocational schools across the province from December 2024 to April 2025.

Vocational students are at a critical stage of self-identity and value formation, facing multiple pressures from academic choices, career planning, and social environments. Their mental health status not only impacts personal development but also relates to educational quality and social stability (Li et al., 2020). This province-wide survey systematically collected data on students’ mental health, lifestyles, and support systems. It aims to reveal key developmental characteristics and potential risk factors within this population, address data gaps in XX’s vocational education sector, and provide evidence-based strategies for schools to establish scientific psychological education systems, refine collaborative support mechanisms, and enhance the effectiveness of moral education. Specifically, this study was guided by the following core hypotheses. The first hypothesis is that sociodemographic factors, specifically the type of primary caregiver, geographic region, place of residence, and perceived family socioeconomic status, significantly differentiate the mental health risk levels of secondary vocational students. The second hypothesis is that unhealthy lifestyle behaviors, including insufficient physical exercise, inadequate sleep, and excessive screen time, are positively associated with higher probabilities of depressive symptoms and mental health risks. However, the detection rate of depressive symptoms is higher among secondary vocational students, which merely reflects intergroup distribution differences. This survey cannot verify any causal relationship between variables.

## Materials and methods

### Study design and participants

This study adopted a mixed-methods design, integrating a large-scale online questionnaire survey with offline thematic interviews. A stratified cluster sampling strategy was employed to ensure representativeness across regions. A total of 54 secondary vocational schools were selected from eastern, western, and northern areas of XX Province, as well as the Pearl River Delta region.

The online survey initially collected responses from 31,010 students. In addition, offline thematic interviews were conducted with 671 students and 197 homeroom teachers and psychological counselors to obtain in-depth qualitative insights.

The details regarding the total number of survey samples are as follows (Table 1):

**TABLE 1:** Clearly specify the initial number of participants, excluded cases, exclusion criteria, the total number of valid cases included in each analysis, and the final total number of participants in the qualitative research phase.

Data category nature of the research	Original participation sample size	Valid after filtering sample size	Sample size exclusion	Data cleaning rate
Online questionnaire	34214	31010	3204	9.36%
Qualitative research	712	671	41	5.76%

Selection criteria explanation:

(1) Completeness.

➀ Missing key variables: If core analytical variables are missing, remove them directly.
➁ Invalid skipping logic: skipped a question but continued answering others, or did not skip any questions but answered key ones incorrectly.

(2) Validity and fraud detection

➀ Answer time:

- Too short: If the text is shorter than “number of words × average reading speed” or the preset minimum reasonable time (5 min), it will be considered invalid.
- Too long: Abnormally long idle time (may be retained depending on circumstances, but requires marking).

➁ Regular answer:

- Linear: Select the same option for all options.
- Wave/snake pattern: For example, a clear pattern of 1-2-3-4-5-4-3-2-1.
- Too low variance: All options in the matrix are highly similar, lacking differentiation.

➂ Trap question/verification question:

- The question explicitly states, “Please select ‘Strongly disagree’ directly.” Candidates who do not choose this option will be eliminated.
- Logical contradiction question involving both preceding and following premises.

(3) Logical Consistency Standard

➀ Demographic logic: For example, the number of children in a family is 10.
➁ Contradictory expressions or options
➂ Quality of open questions
  • Malformed or meaningless characters (e.g., asdfgh, 12345678).
  • Copy and paste the question content as an answer.
  • Advertising, offensive language, or irrelevant content.
  • Too few characters (<5) and not a fill-in-the-blank question.

(4) Outliers

➀ Numerical variable: for example: Age > 120.

Statistical outliers: Identify extreme values using the 3σ rule or a box plot (IQR).

### Inclusion and exclusion criteria

Participants were included if they were officially enrolled students in secondary vocational schools and voluntarily agreed to participate in the study. Questionnaires were excluded if they contained substantial missing data, exhibited response patterns indicative of inattentive answering, or showed logical inconsistencies.

### Measures

The questionnaire covered multiple dimensions, including mental health status, lifestyle behaviors, and social support systems. Standardized measurement instruments were adapted based on established scales widely used in adolescent mental health research. Each scale consisted of multiple items rated on a Likert-type scale.

The internal consistency of the instruments was assessed using Cronbach’s alpha coefficients, which ranged from 0.82 to 0.91, indicating good reliability.

### Data collection procedures

Data collection was conducted between December 2024 and April 2025. The online survey was administered through a secure digital platform under the supervision of trained school staff to ensure standardized implementation. Participation was voluntary and anonymous.

Offline thematic interviews were carried out by trained researchers using semi-structured interview protocols. All interviews were recorded with participants’ consent and subsequently transcribed for analysis.

### Data processing and analysis

All quantitative data were processed and analyzed using IBM SPSS Statistics 28.0 (IBM Corp., United States). The software supported data entry, cleaning, management, and statistical modeling, ensuring the reliability and reproducibility of analyses.

### Statistical analysis

Descriptive statistics, including means, standard deviations, frequencies, and percentages, were calculated to summarize the characteristics of key variables.

Analysis of variance (ANOVA) was conducted to examine differences across multiple groups. When significant effects were identified, post hoc comparisons were performed using the Tukey method to determine specific group differences.

Variable operationalization and analysis of variance (ANOVA): To test the first hypothesis, all variables underwent rigorous operationalization prior to analysis. The independent variables are categorical demographic indicators, including primary caregiver (e.g., parents, grandparents), geographic region (e.g., economically developed regions, eastern regions), residential area (e.g., rural areas, small and medium-sized cities), and perceived household socioeconomic status (ranging from extremely poor to extremely good). The dependent variable is the continuous mental health risk score derived from the General Health Questionnaire. One-way analysis of variance was employed to examine the statistical significance of differences in the mean of the continuous dependent variable across groups defined by different categorical independent variables. By decomposing the total variance into between-group and within-group components, the F-statistic was used to determine whether between-group differences are statistically significant. When a significant difference is detected, the Tukey method is applied for *post-hoc* multiple comparisons to identify specific between-group differences.

In addition, independent samples *t*-tests and correlation analyses were conducted to further explore group differences and relationships among variables. The level of statistical significance was set at *p* < 0.05.

Supplementary note: Significant *p*-values derived from this large sample only indicate statistical differences in group distributions, which do not carry inevitable practical implications for educational practice. Effect sizes and confidence intervals are not reported.

#### Mental health literacy assessment

The second edition of the “China National Mental Health Literacy Questionnaire” released in 2022 by the National Center for Mental Health Assessment at the Institute of Psychology, Chinese Academy of Sciences was adopted. The questionnaire consists of 20 true/false items, comprehensively evaluating respondents’ overall mental health literacy across three dimensions: basic knowledge of mental health, scientific cognitive attitudes, and healthy behavioral patterns. The scoring system assigns 5 points per item, with a maximum total score of 100 points. A total score of ≥ 80 points is defined as meeting the literacy threshold, enabling comparison of mental health knowledge proficiency among different groups. In line with the primary objective of this study, only the true/false module was used for standardized administration, without supplementary self-assessment items.

#### Scales related to mental health status

- General Health Questionnaire (GHQ-12):

This internationally recognized concise mental health screening tool comprises a full 12-item scale, focusing on the respondent’s physical and psychological status over recent weeks. It comprehensively assesses overall mental health risk across four dimensions: depressive mood, anxiety levels, self-confidence, and social functioning. Scores are categorized into three tiers: low-risk, moderate-risk, and high-risk, making it widely applicable for mental health screenings among adolescents and adults.

- Chinese Version of the Second Edition of the Beck Depression Scale (BDI-II):

A mature depression assessment tool comprising 21 items, covering various depressive manifestations such as low mood, negative cognition, social withdrawal, and physical discomfort. It quantitatively evaluates the severity of depressive symptoms and accurately distinguishes students across different levels of depression.

- Adolescent Self-Rating Scale for Insomnia (YSIS):

Comprising 8 items specifically designed for adolescents, this tool assesses insomnia-related manifestations such as frequency of insomnia episodes, sleep-onset difficulty, and sleep maintenance disorders over the past month, quantifying the severity of insomnia.

- Adolescent Self-Injury Specialized Scale:

A self-developed specialized screening scale comprising three items was designed to assess the presence, frequency, and specific methods of self-injurious behaviors, enabling rapid identification of subgroups of vocational high school students exhibiting non-suicidal self-injury behaviors.

- Suicide Risk Scale (SBO-R, Osman’s revised version):

The scale comprises four independent dimensions: past brief suicidal ideation, annual suicide plans and attempted behaviors, verbal suicidal threats, and future suicide likelihood. A total score of 7 or higher is classified as high suicide risk, while a score below 7 indicates low risk. Higher scores correlate with increased probability of suicidal behaviors and are utilized for bulk screening of high-risk students experiencing psychological crises.

#### Measuring tools for family and growth environment

- Social Economic Status Scale (SES):

The study includes six items, collecting basic information such as students’ permanent residential addresses, self-reported family economic status, parents’ educational levels, primary caregivers, and number of children. These data are categorized into distinct family background subgroups for comparative analysis of current conditions.

- Family Function Assessment Scale (APGAR):

A classic family function assessment tool comprising five items, covering five core dimensions: family adaptability, cooperation, growth, intimacy, and emotional acceptance. Scores are used to classify families into three categories: well-functioning families, families with moderate dysfunction, and families with severe dysfunction.

- Station.Kerr (2000) Parent-Child Relationship Questionnaire:

The study comprises a total of 16 items, divided into two dimensions—parent-child relationships (including parent-child and mother-child relationships)—each containing 8 questions. These items assess various aspects of parent-child relationship quality in adolescents, including emotional communication, conflict resolution, and companionship support.

- Self-Reported Growth Trauma Scale:

The scale comprises 13 items covering family background, marital status, early experiences of being left behind or lacking parental care, and prior psychological trauma-related events, designed to identify student samples with early-life growth trauma.

#### Campus interpersonal relationships and campus atmosphere scale

- Campus Atmosphere Questionnaire (Self-designed):

The school’s overall educational environment and psychological atmosphere are evaluated across multiple dimensions, including classroom ambiance, teacher-student interaction, campus inclusivity, and mental health support resources.

- Zou Hong (2006) Simplified Peer Relationship Scale:

Six core items from the original version were selected and categorized into two dimensions—peer acceptance and peer fear—to quantify students’ difficulties in peer interactions and their levels of social anxiety.

- Qu Zhiyong (2002) Simplified Scale for Teacher-Student Relationships:

Eight items were selected, covering four dimensions: intimacy, support, satisfaction, and conflict. Higher scores in the intimacy, support, and satisfaction dimensions indicate a more positive teacher-student interaction, while higher scores in the conflict dimension reflect greater conflicts between teachers and students.

#### Social media use scale series

- Social Media Usage Scale (developed by Deng Yana):

Eight metrics measure the average daily social media usage time and total number of online friends to assess overall social media engagement intensity.

- Online Social Relationship Addiction Sub-questionnaire (developed by Zhou Zhijin and Yang Wenjiao, derived from the Online Addiction Types Questionnaire):

Six items assess students’ tendency toward excessive online socializing and dependence on digital social media.

- Active Social Media Usage Scale (developed by Frison and Eggermont):

There are 5 questions in total, assessing positive online social behaviors such as actively posting updates, sharing content, and proactively contacting friends.

- Passive Social Media Usage Scale (Revised by Liu Qing):

Four items assess negative media usage behaviors such as purposelessly scrolling through news feeds and passively receiving others’ updates; higher scores indicate greater passive engagement.

#### Cognitive and Academic Psychology Scale

- Revised Academic Burnout Scale (MBI-GS, Ni Shiguang 1999):

The study quantifies burnout manifestations among vocational high school students—including disinterest in learning and lack of academic value perception—through three progressive dimensions: emotional exhaustion, mocking attitudes, and low personal achievement sense.

- Meditation Response Scale (RRS):

The scale comprises a total of 21 items, divided into three dimensions: external reflection, compulsive rumination, and depression-related thoughts, measuring the level of cognitive depletion induced by persistent negative thinking.

#### Description of the scale’s reliability and validity

This large-scale online survey has completed reliability testing for all scales. Separate calculations were performed for the overall Cronbach’s α coefficients and internal consistency coefficients across dimensions for each scale in the student, teacher, and parent sample groups. All scale α values exceeded 0.70, indicating strong internal consistency. All scales utilized domestically developed, localized versions whose content validity and structural validity have been validated for adolescent and adult populations. The self-developed specialized screening scales underwent expert review and item optimization following preliminary surveys. KMO tests and Bartlett’s test results demonstrated suitability for this vocational school population sample, confirming stable and reliable measurement outcomes that meet the data collection requirements of large-scale cross-sectional studies (Table 2).

**TABLE 2:** The reliability and validity of the scale were tested.

No.	Scale	Scoring method	Evaluation dimensions	Description
1	Socioeconomic status		1. Gender (Open-ended question)	Select the option that best describes the respondent’s personal circumstances
			2. Date of Birth (Open-ended question)	
			3. Place of Residence	
			4. Your school:	
			5. Your Student ID:	
			6. Primary caregiver (fill in the blank)	
			7. Number of children in your family (including yourself) (fill in the blank)	
			8. Your birth order in the family (fill in the blank)	
			9. Which family members currently live with you? (Select all that apply)	
			10. Mother’s educational level:	
			11. Father’s educational level:	
			12. In your opinion, your family’s socioeconomic status in your area is:	
2	National mental health literacy	Part 1: 28 True/False Questions Part Two: Self-Assessment, 8 four-point scales.	1. Moderate exercise can alleviate mental health issues such as anxiety and depression.	Select the option that best reflects the respondent’s own situation or viewpoint Select the option that best reflects your usual behavior or perspective
			2. The primary cause of most mental health issues lies in genetics.	
			3. Excessive stress and lack of exercise in children are detrimental to brain development.	
			4. To build a child’s self-confidence, parents should frequently praise the child for being smart.	
			5. Increased social interaction among older adults helps slow cognitive decline.	
			6. Emotions such as anxiety and restlessness are harmful and offer no benefits.	
			7. Feeling afraid that something terrible is about to happen	
			8. The earlier mental health conditions are treated, the better.	
			9. With effective treatment, mental health conditions can be alleviated or even resolved.	
			10. Viewing photos or videos of car accidents or disaster scenes may cause psychological trauma.	
			11. Compared to sudden traumatic events, chronic stress has a minimal impact on mental health.	
			12. People who have trouble sleeping at night should catch up on sleep during the day.	
			13. Drinking a small amount of alcohol before bed can help improve sleep quality.	
			14. Being a neat freak means you have OCD.	
			15. Negative emotions can lead to physical illness.	
			16. Hypertension, coronary heart disease, and peptic ulcers are all psychosomatic disorders.	
			17. Online psychological questionnaires can help you determine whether you have a mental health condition.	
			18. It is easy to tell whether someone has a mental or psychological disorder.	
			19. Persistently suspecting you have an illness despite normal medical test results may be a sign of a mental health disorder.	
			20. After medication improves symptoms of a mental or psychological disorder, you can gradually reduce the dosage on your own while monitoring your condition.	
			1. I am confident that I can overcome most difficulties in life.	
			2. When faced with challenges, I can respond rationally and maintain a calm mindset.	
			3. I usually maintain a positive attitude toward life.	
			4. I know how to access information about mental health.	
			5. I know how to seek professional mental health help.	
			6. Mental health has a significant impact on a person.	
			7. Mental health is very important for a person.	
			8. Everyone should learn about mental health.	
3	Social media use	➀The measurement of social media usage intensity is derived from the “Social Media Usage Scale” developed by Deng Yana, which consists of 8 questions. The first two questions assess the number of friends an individual has on social networks and the time spent using them. The remaining five questions employ a 5-point Likert scale [34], with an internal consistency coefficient of 0.784. ➁The measurement of problematic social media use is derived from the “Online Interpersonal Relationship Addiction Subscale” in the “Internet Addiction Type Questionnaire” developed by Zhou Zhijin and Yang Wenjiao. It assesses the extent to which individuals are engrossed in establishing interpersonal relationships online, comprising a total of 6 items [35], with an internal consistency coefficient of 0.834. ➂The measurement of active social media use is derived from the “Active Social Media Use Scale” developed by Frison and Eggermont. It consists of 5 items and measures an individual’s active behaviors on social networks, such as posting photos/videos, updating statuses, and interacting with friends [36], with a Cronbach’s alpha of 0.791. ➃The measurement of passive social media use is derived from the “Passive Social Media Use Scale” revised by Liu Qingqi. This scale consists of 4 items designed to assess the intensity of an individual’s passive reception of information on social networks; a higher score indicates a higher frequency of passive social media use [37], with a Cronbach’s alpha of 0.907.	1. How many contacts do you have on social media (answer based on the social media platform you use most frequently)?	Please select the option that best describes your social media usage
			2. On average, how much time did you spend on social media each day in the past month? (Answer based on your most frequently used social media platform)	
			3. Social media is part of my daily routine	
			4. I feel a little proud when I tell others that I use social media	
			5. Using social media has become a daily habit for me	
			6. When I go without using social media for a while, I feel disconnected from the outside world	
			7. I feel like I’m part of the social media community	
			8. On social media, I update my status (text)	
			9. Uploading photos and videos on social media	
			10. On social media, I actively chat with friends (by sending them messages first)	
			11. Commenting on content posted by friends on social media	
			12. Browsing all my friends’ aggregated updates on social media (Moments, WeChat Recommendations, QQ Feed, etc.)	
			13. On social media, read friends’ status updates and interact with others’ comments	
			14. View text, photos, and videos uploaded by friends on social media	
			15. Browse friends’ profiles on social media (Moments, QQ Zone, Weibo, Douyin homepage, etc.)	
			16. Even though I just logged off, I can’t help but want to keep using social media	
			17. As soon as I wake up in the morning, I want to use social media	
			18. If I can’t use social media, I feel like life is no fun	
			19. My classmates, friends, and family often tell me I spend too much time on social media	
			20. I can spend the entire day on social media without doing anything else	
			21. Because I spend too much time on social media, I find myself neglecting real-life interactions with my family, classmates, and friends	
4	General Health Questionnaire (GHQ-12)	➀The General Health Questionnaire (GHQ-12) was developed by Goldberg D in 1991 to assess the overall mental health of research participants over the past few weeks. ➁Taiwanese scholar Zheng Tai-an developed a 12-item Chinese version of the GHQ based on the original scale and adapted to Chinese cultural characteristics; its reliability and validity have been demonstrated through use in Taiwan. ➂The scale consists of 12 items; selecting 1 or 2 is scored as 0 points, while selecting 3 or 4 is scored as 1 point. The total score ranges from 0 to 12 points. ➃According to previous research, a score of 3 is typically used as a cutoff: a score of 0 indicates a low (or no) health risk, 1–3 indicates a moderate health risk, and a score greater than 4 is considered a high health risk.	(1) Are you able to concentrate when doing things?	Please select the option that best describes your recent health condition
			(2) Have you ever had trouble sleeping due to excessive worry?	
			(3) Do you feel that you are a valuable person?	
			(4) Do you feel you have the ability to make decisions?	
			(5) Do you often feel mentally tense?	
			(6) Do you feel unable to solve problems?	
			(7) Are you able to enjoy your daily activities?	
			(8) Are you able to face the problems you’re facing?	
			(9) Do you feel unhappy and depressed?	
			(10) Have you lost your self-confidence recently?	
			(11) Do you feel like you’re worthless?	
			(12) Do you feel like everything is going smoothly?	
5	Beck Depression Inventory	➀Beck Depression Inventory (Items 1–21) The Chinese version of the Beck Depression Inventory-II (BDI-II) is used. This scale was revised by Beck et al. in 1996 based on the first edition (1961) and is used to assess the severity of each depressive symptom. ➁The scale consists of 21 items across two dimensions: the “Somatization-Affect Dimension” (13 items) and the “Cognitive Dimension” (8 items). Each item is scored on a 0–3 scale, where 0 indicates the absence of symptoms for that item, and 3 indicates pronounced symptoms for that item. A higher total score indicates a more severe degree of depression in the subject (a total score of 0–13 indicates no depression, 14–19 indicates mild depression, and 21–63 indicates moderate-to-severe depression). ➂The scale’s internal consistency coefficient α = 0.94, and the test-retest reliability coefficient is 0.55. For Item 9 of the BDI scale (recent suicidal ideation), a score of 3 is classified as “alert”; a score of 2 as “high risk”; and a score of 1 as “monitoring.” Students identified as requiring early warning should undergo an interview assessment regarding their depression and suicidal ideation.	1. State of Sadness	Based on how you have felt over the past two weeks (including today), select the item from each group that best describes your situation
			2. Regarding Future Prospects	
			3. Sense of Frustration	
			4. Sense of fulfillment	
			5. Guilt	
			6. Expectation of punishment	
			7. Disappointment	
			8 Guilt and self-criticism	
			9. Suicidal thoughts	
			10. Crying	
			11 Anger and rage	
			12 Interest in Others	
			13 Decision-Making Ability	
			14. Concern for Appearance	
			15 Work Status	
			16 Sleep Quality	
			17. Fatigue	
			18. Appetite	
			19. Weight	
			20. Perception of physical health	
			21. Sexual desire	
6	Traumatic Experiences in Childhood	➀Measures the subject’s relatively stable experiences regarding parental marital status, attachment relationships, family functioning, childhood trauma, and coping strategies. ➁Code the responses for each item to calculate the total scale score; a higher total score indicates that the subject’s past experiences were more adverse, and the probability of mental health issues and suicide risk is greater.	1. My parents’ marital status is	Based on your family and life experiences, select the option that best describes your situation
			2. My parents’ relationship	
			3. Before I turned seven, I lived in someone else’s home for more than a week	
			4. I was placed in foster care as a child	
			5. Someone in my family was frequently physically abused	
			6. Someone in my family has a problem with alcohol or drugs	
			7. Someone in my family has been in prison or ran away from home	
			8. I didn’t have enough to eat	
			9. I feel like my parents wish they had never had me	
			10. My parents or caregivers often humiliate me	
			11. I have been severely beaten, and people around me noticed	
			12. Someone has ever threatened or coerced me into doing sexual acts	
			13. I have visited a hospital’s psychiatric, psychological, or sleep medicine department:	
7	Self-Rating Insomnia Scale: YSIS	➀Calculate the total score; a higher score indicates more severe insomnia ➁Categories are as follows: < 22, normal; 22–25, mild insomnia; 26–29, moderate insomnia; ≥ 30, severe insomnia.	1. Over the past 2 weeks, how has your overall sleep quality been?	Based on your sleep situation, select the option that best describes your personal circumstances
			2. Overall, how satisfied have you been with your sleep over the past 2 weeks?	
			3. Over the past 2 weeks, have you had difficulty falling asleep after going to bed?	
			4. Over the past 2 weeks, have you woken up frequently during the night:	
			5. Over the past 2 weeks, have you woken up early and had trouble falling back asleep?	
			6. Over the past 2 weeks, have you felt sleep-deprived?	
			7. Over the past 2 weeks, have you still felt tired upon waking in the morning?	
			8. Over the past 2 weeks, has your sleep affected your daytime functioning:	
			9. Over the past 2 weeks, how many hours of sleep have you gotten each day on average:	
			10. Have you had sleep problems in the past:	
8	Family Function: Family APGAR	➀Designed in 1978 by Smilkstein at the University of Washington in Seattle, USA, and localized into Chinese in 1995 by Lü Fan et al. ➁3-point scoring system (A = 2, B = 1, C = 0): “Often” scores 2 points, “Sometimes” scores 1 point, and “Rarely” scores 0 points. ➂Add the scores from the five questions to obtain the total score. A total score of 7–10 indicates good family functioning; 4–6 indicates moderate impairment; and 0–3 indicates severe impairment.	1. I am satisfied that I can turn to my family for help when I encounter difficulties.	Based on the respondent’s family situation, select the option that best describes their family.
			2. I am satisfied with the way my family discusses matters and shares problems with me.	
			3. I am satisfied that when I want to engage in new activities or pursue new directions, my family accepts and supports me.	
			4. I am satisfied with the way my family expresses their feelings toward me and responds to my emotions (such as anger, sadness, or love).	
			5. I am satisfied with the way my family interacts with me.	
9	Physical Activity Habits Scale	➀The minimum total score for the scale is 14 points (the participant selects “Completely Disagree” for all 14 items), and the maximum score is 70 points (the participant selects “Completely Agree” for all 14 items). ➁Scores are divided into 4 intervals: First interval, scores equal to or greater than 14 points (1 × 14 = 14), less than or equal to 28 points (2 × 14 = 28); Second interval, greater than 28 points, less than or equal to 42 points (3 × 14 = 42); Third interval, greater than 42 points, less than or equal to 56 points (4 × 14 = 56);Fourth range: 56 or higher, but less than 70 (5 × 14 = 70). ➂A total score in the first range indicates that the subject never engages in physical exercise; in the second range, they engage in physical exercise occasionally;in the third range, they are beginning to engage in regular physical exercise, but their exercise behavior is inconsistent—sometimes proactive, sometimes reactive, with varying frequency and intensity—and they are prone to interrupting or abandoning exercise due to minor difficulties or distractions; in the fourth range, they consistently and proactively engage in physical exercise over the long term, demonstrating strong willpower and sustained, stable exercise behavior (having developed a habit of physical exercise). The higher a participant’s total score falls within the fourth interval, the stronger their exercise habit is indicated to be.	1. To date, I have been able to consistently engage in physical exercise	Based on your exercise habits, select the option that best applies
			2. I have exercised regularly over the past 6 months	
			3. Physical exercise has become an important part of my daily life	
			4. Generally speaking, I engage in physical exercise on most days of the month	
			5. I exercise at least three times a week	
			6. Since I started exercising regularly, I have rarely gone more than a week without exercising	
			7. I usually exercise on a regular schedule	
			8. When exercising, I flexibly decide the time, location, activities, and intensity based on actual circumstances	
			9. I am able to balance physical exercise with other important matters	
			10. I have learned and mastered a physical exercise routine that suits me	
			11. Through regular exercise, I have improved my physical fitness, toned my body, and enhanced my adaptability	
			12. Through regular exercise, I have strengthened my willpower, boosted my self-confidence, and improved my mental state	
			13. I have personally experienced how physical exercise helps me maintain good physical condition	
			14. I have fully experienced the joy of physical exercise	
10	Ruminative Response Scale	Likert 4-point scale: 1–4 ➀Symptom rumination: 1, 2, 3, 4, 6, 8, 9, 16, 17, 18, 21; ➁Brooding: 5, 10, 13, 14, 15; ➂Reflective Pondering: 7, 11, 12, 19, 20 ➃Yang Juan, Ling Yu, Xiao Jing, & Yao Shuqiao. (2009). Analysis of preliminary results from the Chinese version of the Rumination Response Scale among high school students. Chinese Journal of Clinical Psychology (01), 27–28+31.	1. Thinking about how lonely I am	Respondents should select the option that best describes whether they have these thoughts and behaviors when feeling sad or depressed, and to what extent
			2. Thinking that since I feel terrible, I’ll surely struggle to get today’s work done	
			3. Reflecting on my feelings of fatigue and pain	
			4. Realizing it’s hard to concentrate	
			5. What did I do to deserve this?	
			6. I feel so negative and defeated	
			7. Analyzing recent events to try to find the cause of my low spirits	
			8. Realize that I seem to have lost all feeling toward anything	
			9. Thinking, “Why can’t I just get started?”	
			10. I wonder, “Why do I always react this way?”	
			11. Walk away alone to think about why you feel this way	
			12. Write down your thoughts and analyze them	
			13. Recall recent situations, hoping things have improved	
			14. Think, “Why can’t I do things a little better?”	
			15. Thinking, “Why can’t I do things a little better?”	
			16. Reflect on how sad you feel	
			17. Reflect on all your failures, shortcomings, mistakes, and missteps	
			18. Reflecting on how I can’t do anything right	
			19. Analyzing my personality to try to find the cause of my discouragement	
			20. Going somewhere alone to think about your feelings	
			21. Feeling angry just thinking about yourself	
11	Academic Burnout	➀This scale was revised by Ni Shiguang (1999) from the MBI-GS (Yang Huizhen revision) ➁The scale is divided into three dimensions: emotional exhaustion (1, 2, 3, 4), cynicism (8, 9, 14, 15), and low personal accomplishment (5, 7, 10, 11, 12, 16), ➂The scale consists of 14 items (total score 14–98); a higher score indicates a higher degree of academic burnout. ➃There are two criteria for determining results: 1) Based on the mean score of the academic burnout subscale, divide the sample into the top 27% and bottom 27% of scorers (Ni Shiguang, 1999) 2) Based on the data at the time, independently define a cutoff of 56 points on the academic burnout scale and a mean score of 4 as the criteria for assessing academic burnout among college students (Ju Song, 2011)	1. Schoolwork is draining me mentally	Respondents selected the option that best matched their current feelings about their studies
			2. After a day of classes, I feel completely exhausted	
			3. When I wake up early in the morning and think about having to go to class again, I can’t muster the energy	
			4. Being busy with schoolwork all day leaves me feeling mentally strained	
			5. I can efficiently tackle academic challenges	
			6. I feel I can make a significant contribution to the school	
			7. Since entering this major, I’ve become less and less interested in the coursework	
			8. I’m becoming less and less enthusiastic about the school curriculum	
			9. I believe I can do well on my schoolwork	
			10. I feel a sense of satisfaction when I complete certain assignments	
			11. I have done many meaningful things at school	
			12. I care less and less about whether my studies have any meaning	
			13. I doubt the importance of my studies	
			14. Academically, I feel confident and efficient in completing my work	
12	Parent-child relationship	➀The Parent-Child Relationship Questionnaire developed by Station and Kerr (2000) was used, consisting of 16 items—8 for father-son relationships and 8 for mother-son relationships. Scoring method and interpretation of scores ➁Item Scoring: A 3-point scoring scale was used: “Never” = 1 point, “Sometimes” = 2 points, “Often” = 3 points. ➂Reversed-scoring items: Items 1, 3, 4, 7, 9, 11, 12, and 15 ➃Total Score: Calculate the average scores separately for father-son and mother-son relationships; higher scores indicate a better relationship.	1. Are you disappointed in your father?	Respondents should select the option that best describes their relationship with their father Respondents should select the option that best describes their relationship with their mother
			2. Do you and your father understand each other?	
			3. Do you wish your father would change?	
			4. Do you argue or fight with your father?	
			5. Are you proud of your father?	
			6. Do you accept your father’s way of doing things?	
			7. Has your father ever made you angry or upset?	
			8. Does your father support and encourage you?	
			1. Are you disappointed in your mother?	
			2. Do you and your mother understand each other?	
			3. Do you wish your mother would change?	
			4. Do you argue or fight with your mother?	
			5. Are you proud of your mother?	
			6. Do you accept your mother’s way of doing things?	
			7. Has your mother ever made you angry or upset?	
			8. Does your mother support and encourage you?	
13	Self-Harm Questionnaire (Jump to Questionnaire)		1. Have you ever intentionally harmed yourself without intending to die? For example, cutting your body with a sharp object or taking an overdose of medication. [Multiple choice]	Self-harm refers to intentionally harming oneself, but does not include self-harm motivated by suicidal intent.
			2. How many times have you engaged in self-harm in the past 12 months? [Single choice]	
			3. Please estimate the number of times you have engaged in the following self-harm behaviors in the past 12 months (e.g., 0 times, 10 times, 100 times, etc.) [Multiple choice]	
14	BSQ-R Suicide Scale (Go to Questionnaire)	Adapted by American psychology professor Osman et al., the test results can be used to identify individuals at risk of suicide and specific risk behaviors. The four independent items of the BSQ-R represent different dimensions of suicide. ➀The first item, “Have you ever thought about or attempted suicide ➁”, is used to assess an individual’s past transient suicidal ideation, suicide plans, and suicide attempts ➂The second item, “Have you thought about or attempted suicide in the past year?”, determines the incidence of suicidal ideation over the past 12 months; ➃The third and fourth items are used to measure the subject’s suicide threats and self-reported likelihood of future suicide, respectively. ➄The total score ranges from 3 to 18 points; a higher score indicates a higher risk of suicidal behavior. Typically, a score of 7 or higher is classified as indicating a high risk of suicidal behavior, while a score below 7 is considered to indicate a low risk.	1. Have you ever thought about or attempted suicide?	Respondents should select the option that best reflects their actual situation
			2. How often have you thought about suicide in the past year?	
			3. Have you ever told someone that you were going to commit suicide or might commit suicide?	
			4. How likely are you to attempt suicide in the future?	
15	Teacher-student relationship	Items 1–3 are scored positively; items 4–5 are scored negatively	1. I feel comfortable sharing my innermost thoughts with my homeroom teacher.	Select the option that best reflects the respondent’s experience with their teacher over the past 6 months
			2. When I encounter difficulties in my studies, my teacher patiently explains them to me.	
			3. I am satisfied with my relationship with my homeroom teacher.	
			4. When I am sad or feel wronged, my teacher does not take the initiative to show concern for me.	
			5. My teacher treats me unfairly.	
			6. When you have something on your mind, who do you turn to first?	
16	Campus Atmosphere Questionnaire	Statistical analysis of the qualitative frequency of various behaviors	1. There are students fighting at our school	
			2. There are students stealing at our school	
			3. There are students at our school who smoke and drink	
			4. There are students at our school who extort, threaten, or bully others	
			5. Some students at our school are unmotivated to study, skip school, or skip classes	
			6. There are students at our school who cheat on exams	
17	Peer relationships	Questions 3 and 4 are reverse-scored; questions 1–4 measure peer acceptance, while questions 5–8 measure fear of peers	1. I find it easy to make friends at school	Respondents should assess how closely the descriptions match their own experiences and select the option that best applies to them
			2. I always enjoy spending time with my classmates	
			3. No one in my class talks to me	
			4. When I’m upset or troubled, I can’t find a classmate to talk to	
			5 I only talk to friends I know very well	
			6. I worry about what other classmates might say about me	
			7. I feel shy when I’m surrounded by classmates I know	
			8 I feel nervous talking to classmates I don’t know	

#### Supplementary note on coding standards and mixed-method interpretation

1. Relevant themes, logic, and procedures for qualitative research

Qualitative research employs three thematic questions and analyzes corresponding response data statistically to conduct qualitative analysis, covering the following topics:

(1) Research topic 1: key aspects of students’ psychological development

- Question: What aspects of your academic life during secondary vocational education did you focus on?
- Logical positioning: Aims to collect students’ current focus points without preconceptions, identifying what holds primary significance in their “lifeworld.”
- Process function: Identify high-frequency terms (e.g., interpersonal relationships, academic stress) to lay the groundwork for identifying key pain points.

(2) Research Topic 2: sources of psychological developmental disorders in students

- Question: What obstacles or difficulties have you encountered during your personal growth and development?
- Logical Positioning: After identifying the focal points, this step transforms surface-level concerns into deeper challenges by examining specific “barriers” and “difficulties,” aiming to pinpoint the concrete obstacles hindering student development.
- Process function: Transform “focus areas” (e.g., learning) into specific “barriers” (e.g., difficulty understanding lectures, academic pressure), and attribute them to underlying causes (e.g., family conflicts, management disputes).

(3) Research theme 3: effective experiences from a student’s perspective

- Question: When you or a classmate is experiencing psychological distress, what forms of support do you consider effective?
- Logical approach: After identifying pain points, shift to a “strength-based perspective” and ask students what they believe can address these issues within their experience. This not only helps gather insights but also reveals inherent support systems (such as peers or informal activities).
- Process approach: shift from a “problem-oriented” to a “solution-oriented” approach, and identify the support resources available to students.

The core logic of the three aforementioned themes and their corresponding questions follows this sequence: “current situation perception (focus point) → obstacle identification (pain points) → solution pathways (best practices),” forming a typical closed-loop problem-solving approach.

2. Key points of “coding” in qualitative research

The coding mechanism of this survey primarily comprises the following four key operational components:

(1) Key point 1: keyword extraction and classification

The review team will extract core vocabulary from students’ spoken expressions and categorize it under higher-level concepts.

Document evidence:

- Original responses (oral): “My roommates keep stealing my things,” “The three of them collude to exclude me,” “I’m being slandered by classmates behind my back.”
- encoded results: “Dormitory argument,” “Small-group isolation,” “Verbal ridicule”
- Analysis: The evaluation mechanism identified these specific incidents as falling under the category of “interpersonal conflicts” and classified them as “classmate disputes.”

(2) Key point 2: frequency statistics and quantitative conversion

Convert qualitative data into quantitative data (review results). Each occurrence of a specific category keyword increments the category counter by 1.

Document evidence:

- The 30.61% share attributed to “interpersonal relationships” was not derived from questionnaire scores, but rather calculated by counting the frequency of all responses categorized as “interpersonal relationships.”
- Analysis: By calculating the frequency of various codes, the evaluation mechanism identified “interpersonal relationships” as the most prominent concern among students (30.61%), and thus designated it as the primary focus in the report.

(3) Key point 3: subitem splitting (multi-dimensional coding)

The review team did not simply treat each response as a single data point; instead, they adopted a “one-to-many” approach, where complex responses could be broken down and assigned to different coding categories.

Document evidence:

- Break down answers to multi-dimensional questions into separate sub-items (for example, split “high academic pressure and frequent exclusion by roommates” into two distinct items: “academic pressure” and “interpersonal relationships”).
- Analysis: This encoding method ensures data saturation, but results in the original 671 records being split into 1,292 valid responses.

(4) Point 4: anchoring with a typical example

To prevent encoding from becoming overly subjective or distorted, the review mechanism includes “typical examples.” In addition to counting frequency, it retains the most representative original statements for each encoding category to verify the encoding.

Document evidence:

- Table 3 lists specific sentences for each coding category (e.g., “learning ability anxiety”), such as “I can’t remember the physics formulas at all.” Therefore, Table 3 in qualitative research should include additional examples as shown below.
- Analysis: The review mechanism does not operate in isolation from context during the “coding” process. A typical example is that review criteria serve as key references for determining the appropriateness of coding.

**TABLE 3:** Summary of typical examples for each coding category.

Type	Main keywords	Quantity	Proportion	Typical Example
Anxiety about learning abilities	Subject bias, difficulty understanding lectures, weak foundational knowledge, and poor learning methods	287	22.21%	I can’t remember physical formulas at all; I forget English words once I’ve memorized them; and I’m struggling to keep up with the pace of learning mathematical functions.
The pressure of entrance examinations	Vocational college entrance exams, certification examinations, internship assessments, and academic rankings	243	18.81%	The exams for Chinese, Mathematics, and English are coming up next semester. Is it still too late to start reviewing? Failure to pass the certification exams may affect your further education opportunities.
Conflicts among classmates	Dormitory arguments, group isolation, verbal ridicule	189	14.63%	My roommates always steal my things without admitting it; the three of them work together to exclude me, and my classmates spread negative rumors about me behind my back.
Daily academic workload	Excessive homework, frequent exams, and prolonged evening study sessions.	121	9.37%	Doing homework until 12 PM every day and taking three exams per week is extremely exhausting; the evening study sessions also run from 7 PM to 10 PM.
Phone control conflict	Recover phones, block games, and impose communication restrictions	95	7.35%	We’re required to turn off our phones during evening study sessions, but we still need to use them for research. We’re also not allowed to play games on weekends—it’s really frustrating. My parents always check my chat records.
Dissatisfaction with campus management	Exercise regimen, cafeteria quality, dormitory conditions	87	6.73%	Starting morning exercises at 6 a.m. is too early; it’s difficult to wake up. The food in the cafeteria is salty and expensive, and there’s no air conditioning in the dormitory. During summer, it’s so hot that you can’t sleep.
Family conflict	Parental arguments, financial control, and excessively high expectations	76	5.88%	My parents argue constantly every day, creating a very oppressive atmosphere at home. I want to buy some study materials, but my pocket money isn’t enough. Moreover, they insist that I choose the accounting major—which I really don’t like.
Social phobia	Lack of initiative in initiating conversations, nervousness in public settings, fear of evaluation	63	4.88%	I hesitate to speak during group discussions, take detours from teachers when passing by them, and worry that others might think I’m strange.
Student relation	Teacher bias, poor communication, criticism damaging self-esteem	58	4.49%	The teacher only paid attention to students with good grades; during class, he always asked me questions specifically, and the homeroom teacher publicly scolded me until I cried.
Anxiety about one’s physical appearance	Obesity, acne, worsening myopia, developmental issues	42	3.25%	Perceiving oneself as overweight, avoiding wearing skirts; experiencing recurrent forehead acne that prevents social interactions; and requiring increasingly higher prescription strength for glasses.
Economic pressures	Insufficient pocket money, being scammed while working a part-time job, rising prices	31	2.40%	My monthly pocket money is only 300 yuan—insufficient to cover daily expenses. I also earn income from part-time flyer distribution, but my wages are often delayed. Moreover, after the cafeteria raised prices, I can no longer afford meals there.

3. Explanation of the “qualitative research evaluation mechanism”

1. Complete workflow:

(1) Collection: Gather written records from workshops and interviews (original corpus).
(2) Clean: Remove invalid data (e.g., irrelevant responses or incomprehensible spoken language).
(3) Open coding: Read the text and extract keywords (e.g., “depression,” “cell phone,” “parental argument”).
(4) spindle code: Keyword classification. For example, classify “depression” and “anxiety” under “mental health”; classify “mobile phone control” and “receiving mobile phones” under “conflict management.”
(5) Selective Coding and Splitting: Identify core categories (e.g., the 11 types in Table 2) and split composite responses into sub-items.
(6) Statistics and Validation: Calculate the frequency for each category and select the most representative “typical examples” for qualitative validation.

2. Review mechanism application:

(1) Collection and transcription of open-ended questions:

The first step in the review process involves recording and transcribing open-ended responses from workshops and interviews. The frequent appearance of “typical examples” (e.g., “My roommate always steals my things,” “My parents argue every day”) in the documents demonstrates that the review mechanism preserves the “authenticity” and “contextuality” of the original data.

(2) Content analysis method and coding classification:

- Keyword extraction: The review mechanism extracts keywords from collected text. For example, under “Sources of barriers,” various responses are categorized into terms such as “learning ability anxiety” and “exam pressure.”
- Frequency statistics: The evaluation mechanism counts the occurrence frequency of keywords. A response may include multiple dimensions (with sub-items split), and counts are performed across all dimensions.
- Category Analysis: Group scattered responses into themes. For example, combine “not daring to speak up” and “fearing judgment” into “social anxiety.”

(3) Triangle verification mechanism:

The document in “Effective Experiences from the Student Perspective” presents a cross-sectional comparative analysis of students’ qualitative responses against those from the “teacher perspective.” The evaluation mechanism incorporates “cross-group validation,” which assesses data validity and depth by comparing cognitive differences between teachers and students.

(4) Quantitative conversion:

The evaluation mechanism quantified qualitative data. For example, it calculated the proportion of responses by category (e.g., “interpersonal relationships” accounted for 30.61%).

4. How can qualitative findings supplement or interpret the analysis of quantitative results?

While qualitative research focuses solely on vocational students in selected cities within Guangdong’s Pearl River Delta region—including Guangzhou, Shenzhen, Zhongshan, Foshan, and Qingyuan—quantitative studies cover a broader geographical area. Due to constraints such as time and resource limitations, the design of qualitative research questions can only address certain core issues within this framework. Consequently, the target samples and data obtained through qualitative research cannot strictly or specifically interpret all findings from quantitative studies; they serve primarily as macro-level references for partial trends. To meet the rigorous requirements of inferential research designs, further in-depth investigations through dedicated mechanisms are necessary for supplementation.

The following is a partial inference analysis table (Table 4):

**TABLE 4:** The following is a partial inference analysis table.

Quantitative results	Qualitative results	The complementary/interpretive role of qualitative analysis in quantitative studies
High detection rate of depression (26.35%) The presence of self-harm/suicide thoughts	Research Theme 1: 30.61% focused on “interpersonal relationships,” while 25.51% focused on “academic pressure.” Research Theme 2: 50.39% of the issues stem from academic-related challenges (e.g., difficulty understanding material or failing exams).	Uncovering underlying causes: Quantitative data indicate the presence of a disease, while qualitative data reveal its etiology. Students’ depression is not unfounded; rather, it stems from the academic pressure of preparing for the higher education entrance exam (even though they attend vocational schools but aspire to pursue further studies) and issues related to dormitory conflicts or social isolation within small groups.
Low mental health literacy (4.3%) There are widespread misconceptions.	Theme 3: 26.7% found “emotional support” (listening, companionship) to be effective; 25% considered “attention diversion” (eating delicious food, playing games) effective. Only 5.3% mentioned “self-regulation.”	Explain the behavioral logic: Why is literacy low? Because students seek help primarily through peer support and real-life contexts. When encountering problems, they tend to confide in friends or vent through gaming rather than seeking professional psychological counseling. This explains why quantitative data indicate a lack of awareness regarding “professional help-seeking.”
Low family function assessment score (77.37%) Teacher-student relationship: Low willingness to actively express concerns	Topic 2: 5.88% of barriers stem from “family conflicts” (parental arguments, excessively high expectations). Topic 3: Students identify that effective support primarily comes from “peers” and “informal activities.”	Revealing misalignment in support systems: Quantitative data indicate poor family functioning, while qualitative data specifically identify “parental control tendencies” and “emotional neglect” as contributing factors. This explains why students, when facing psychological distress (quantitatively: reluctance to seek teachers’ help), are more inclined to confide in peers (qualitatively: peers serve as their primary support source), since families and schools (teachers) often act as sources of stress or lack credibility.
Academic burnout (49.19 points)	Topic 2: 22.21% experience “anxiety about learning ability,” while 18.81% face “pressure from college entrance exams.” Typical examples: “I can’t remember the physics formulas,” “I didn’t pass the skill certification exam.”	Specific sources of burnout: Quantitative data represent outcomes, while qualitative data reflect processes. Students are not merely “lazy,” but rather exhibit “weak foundational knowledge” (inability to comprehend material) and “single-minded, high-pressure goals” (the imperative to obtain certifications or pass the college entrance examination). This imbalance between “high pressure and low competence” specifically elucidates the etiology of burnout.

## Results

**FIGURE 1:**
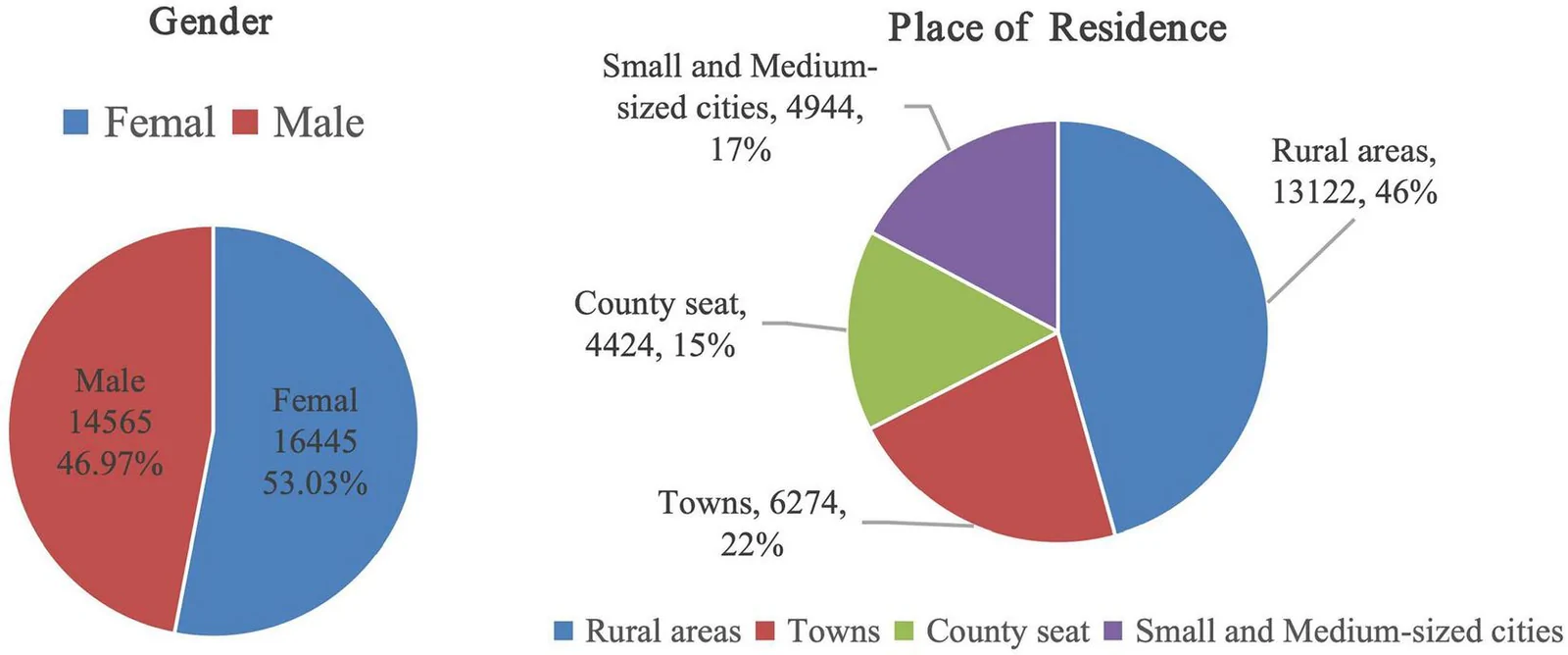
Student sample characteristics: gender and residence.

**FIGURE 2:**
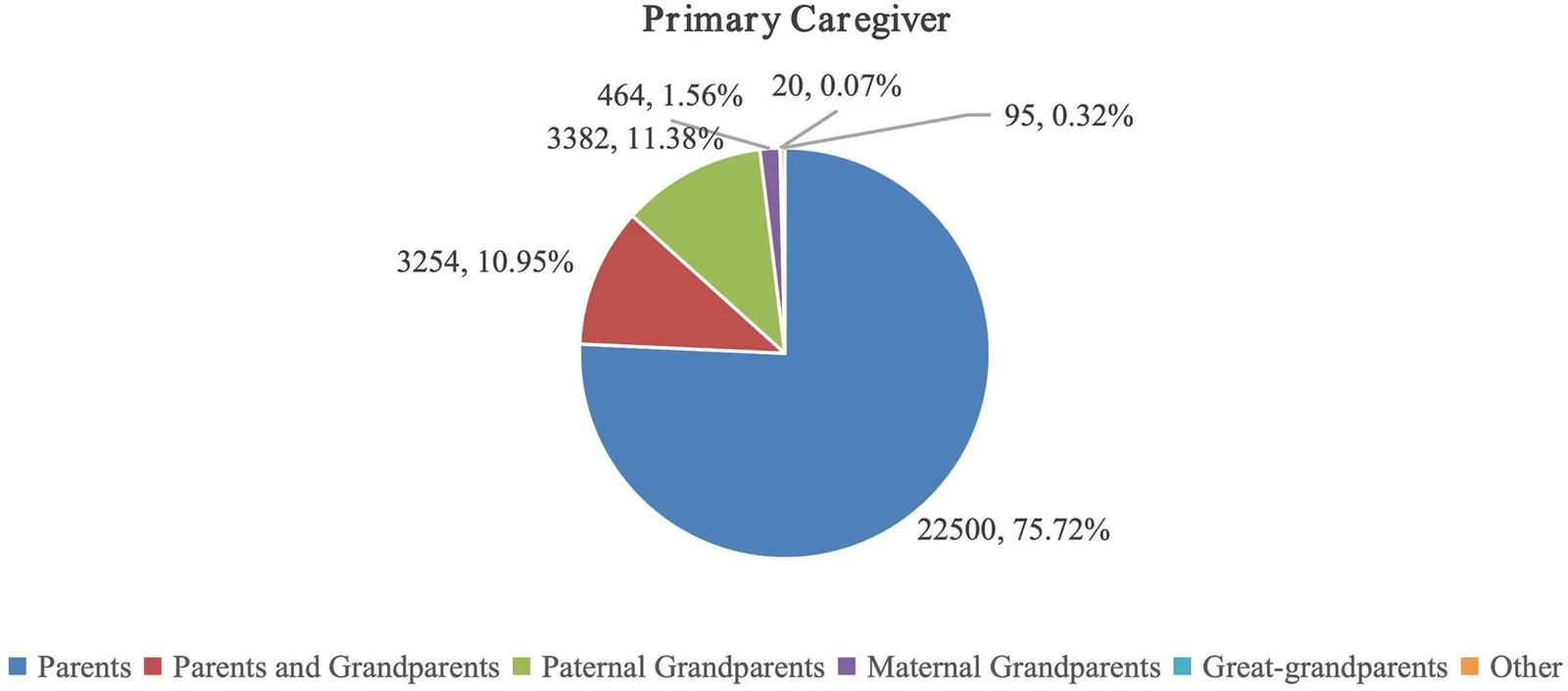
Student sample characteristics: primary caregiver.

**FIGURE 3:**
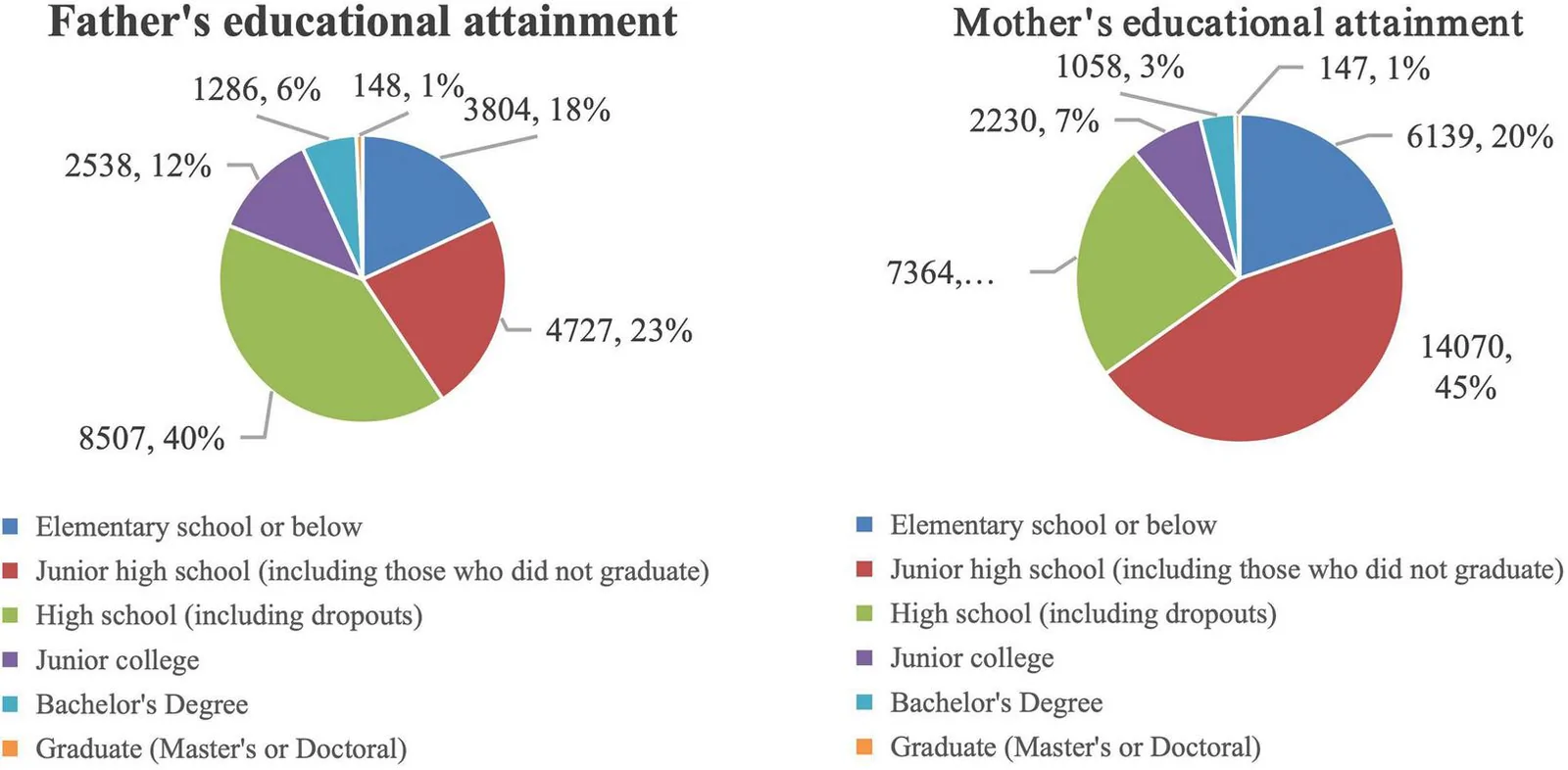
Student sample characteristics: parental educational attainment.

Among the student sample (Figure 1), females accounted for 53.03% (16,445 individuals), while males made up 46.97% (14,565 individuals). The age range was concentrated between 16 and 18 years old. Rural and township household registrations accounted for 62.55% (19,397 individuals) of the total sample. There were only 3,471 only children (11.22%), and the proportion of non-only children was 88.78% (27,531), and the phenomenon of intergenerational upbringing (including either one or both of the grandparents) accounted for 13.01% (4,034) (Figure 2). A significant proportion of parents had an education level of junior high school or below (accounting for 59.76%) (Figure 3).

Overall characteristics indicate that vocational students commonly face structural challenges such as “weak family support, heavy economic pressure and limited access to resources are prevalent. Meanwhile, most parents have a low educational level, and a large proportion of the subjects come from rural and township areas.” Providing crucial contextual variables for understanding mental health risk distribution and psychological needs patterns.

### Survey data presentation and results analysis

#### Analysis of online questionnaire survey

The student online questionnaire explored multiple dimensions including mental health literacy, mental health status, lifestyle, social relationships, and academic burnout. Key findings are as follows:

#### Students’ mental health literacy requires improvement

**FIGURE 4:**
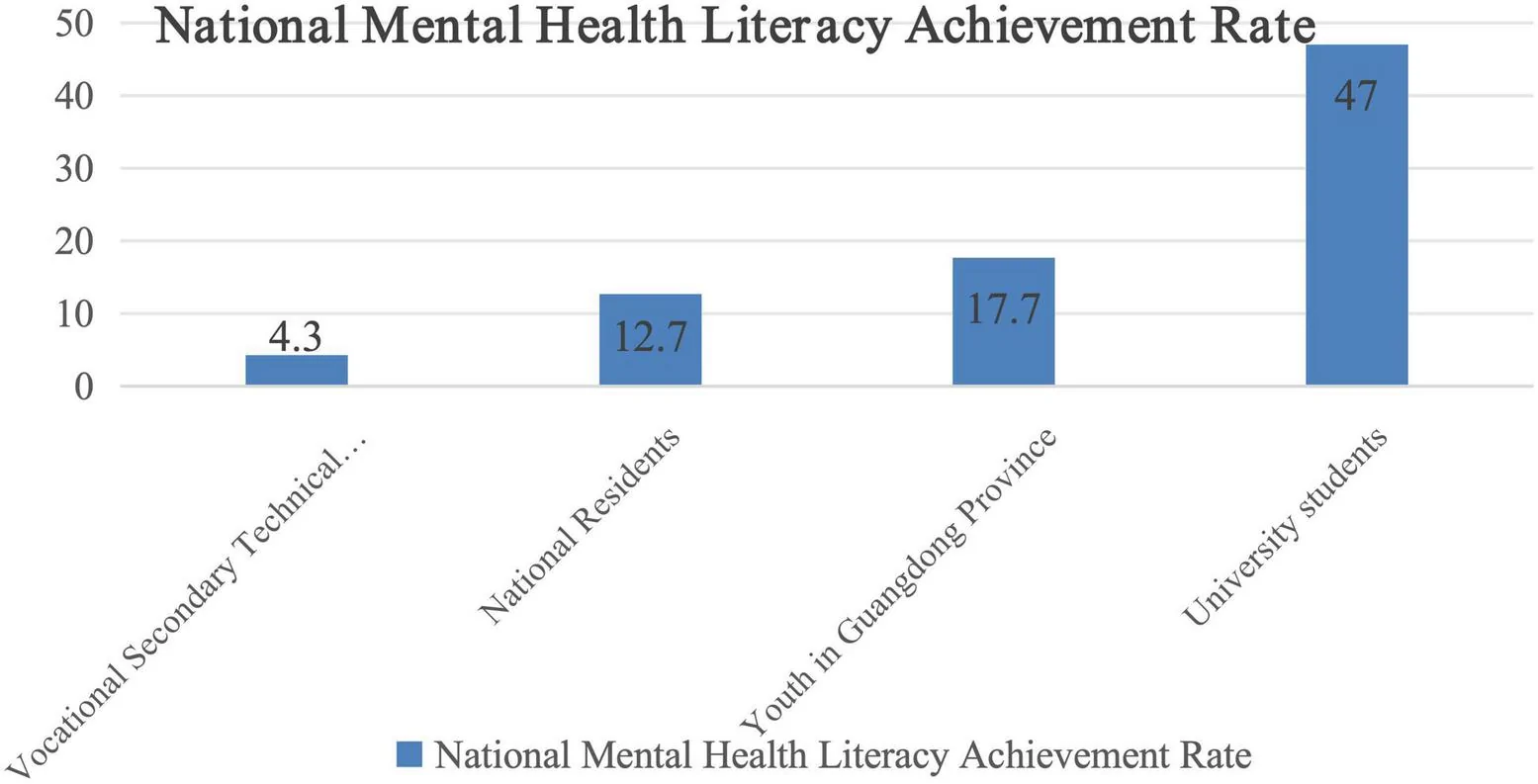
Comparison of national mental health literacy achievement rates.

**FIGURE 5:**
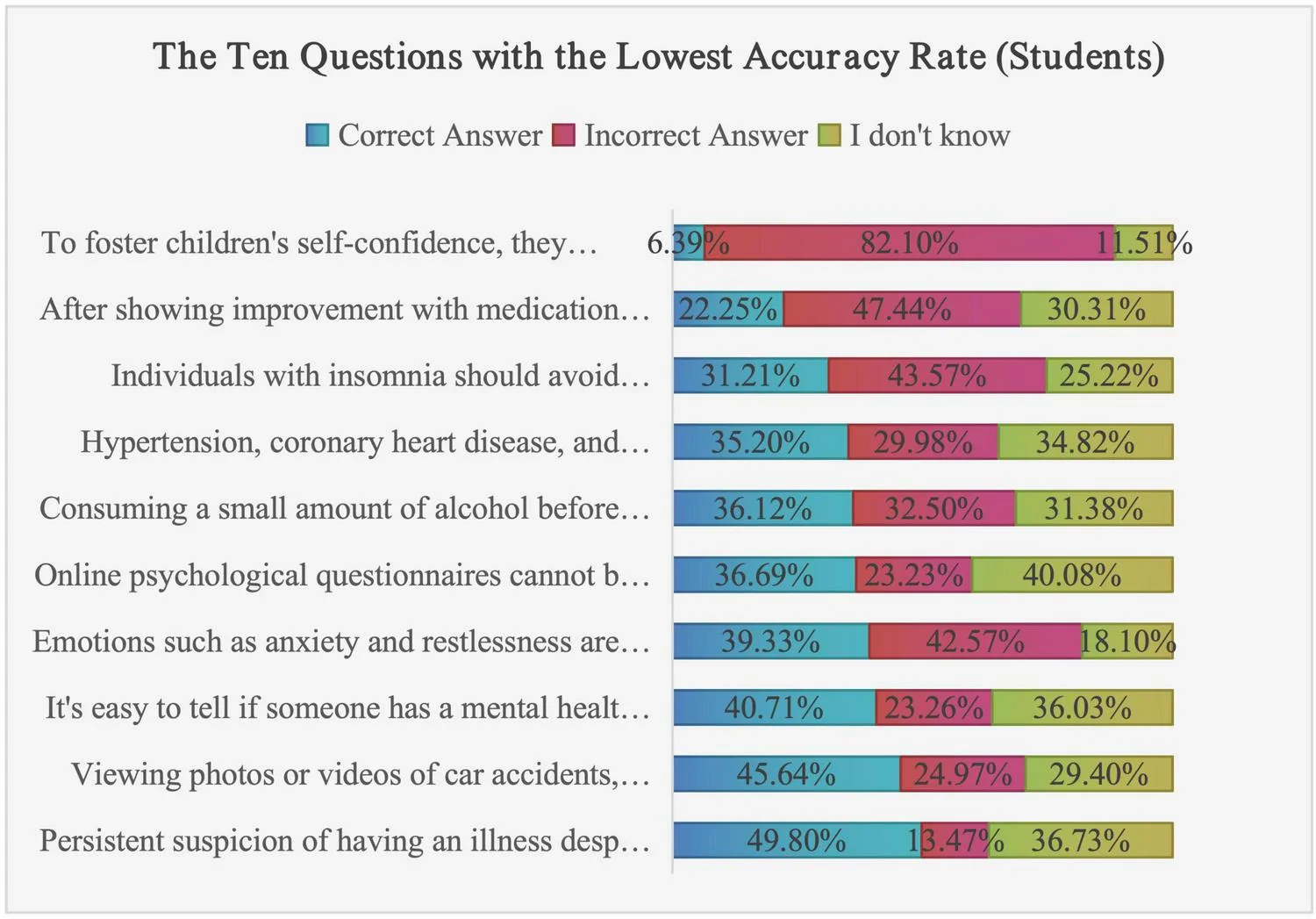
Top 10 questions with lowest accuracy rates in mental health literacy.

Only 4.3% of students met the benchmark for mental health knowledge, below the national average (12.7%), (Figure 4). Major misconceptions centered on mental illness treatment, sleep health, and educational methods. For instance, only 36.1% correctly identified “drinking alcohol before bed to aid sleep” as incorrect, while fewer than 7% correctly understood that “praising children for being smart” is harmful (Figure 5). This indicates students’ mental health cognition remains largely experiential, lacking scientific frameworks and protective awareness.

#### Mental health indicator detection rates

**FIGURE 6:**
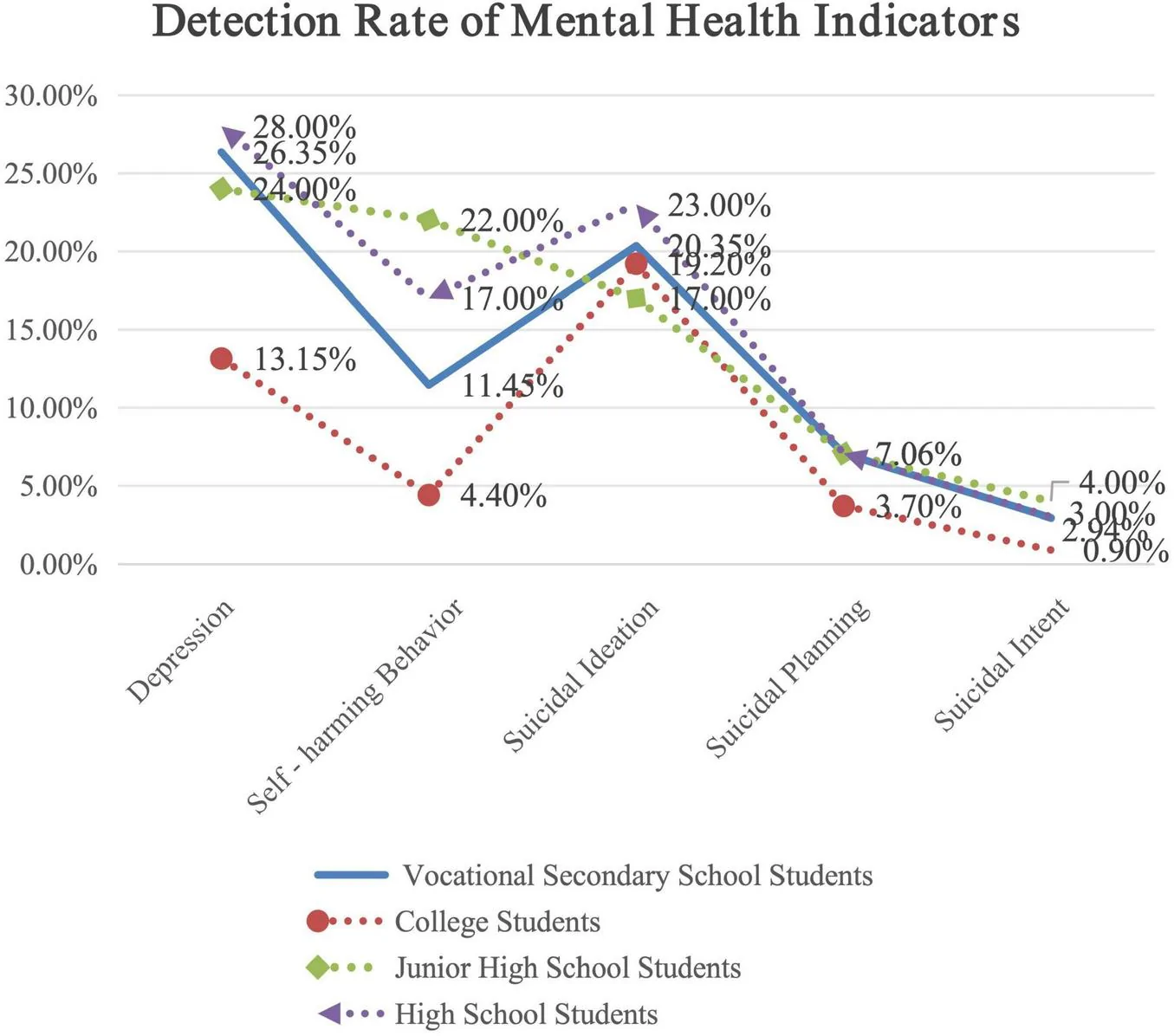
Comparison of mental health indicator detection rates among vocational students (Bachman et al., 2024).

**FIGURE 7:**
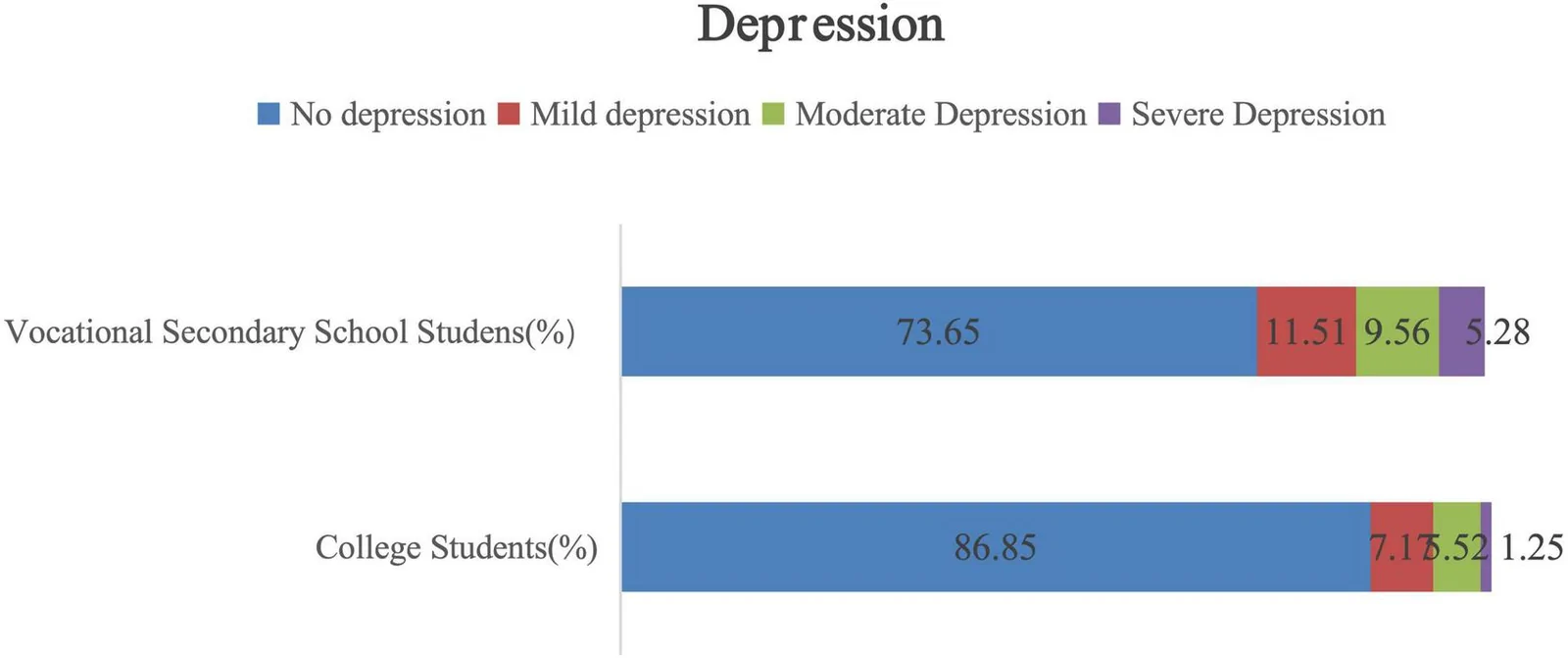
Comparison of depression rates among secondary vocational students and college students (Data on college students were obtained from the 2025 mental health survey of a key undergraduate university in XX Province.).

Data indicates that (Figure 6) vocational students exhibit depression detection rates of 26.35%, self-harm detection rates of 11.45%, and suicidal ideation detection rates of 20.35%—figures comparable to secondary school students but noticeably higher than university students for depression and self-harm behaviors (Figure 7). Mild depression accounts for 11.5%, moderate for 9.6%, and severe for 5.3%.

#### Factors influencing students’ mental health levels

**FIGURE 8:**
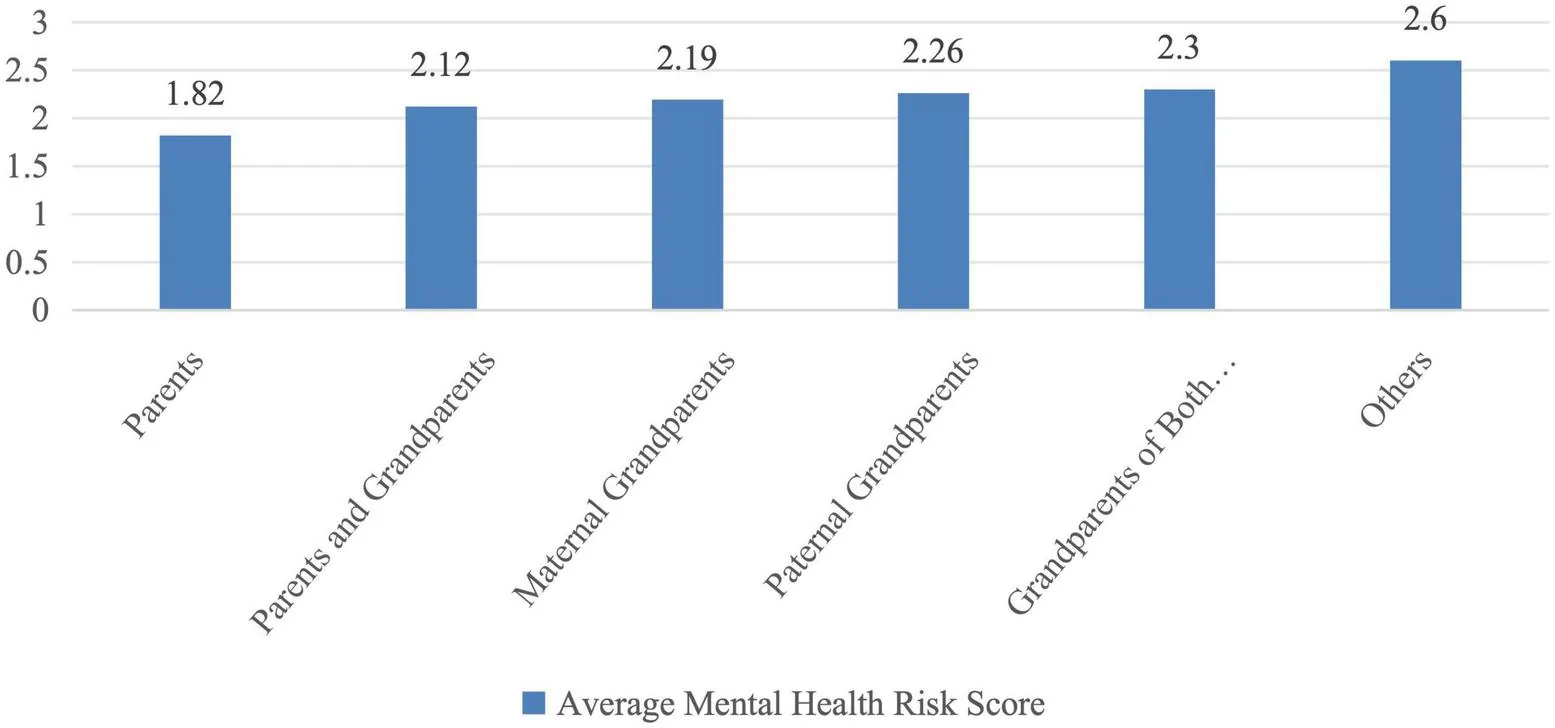
Differences in mental health risk scores among different types of caregivers.

**FIGURE 9:**
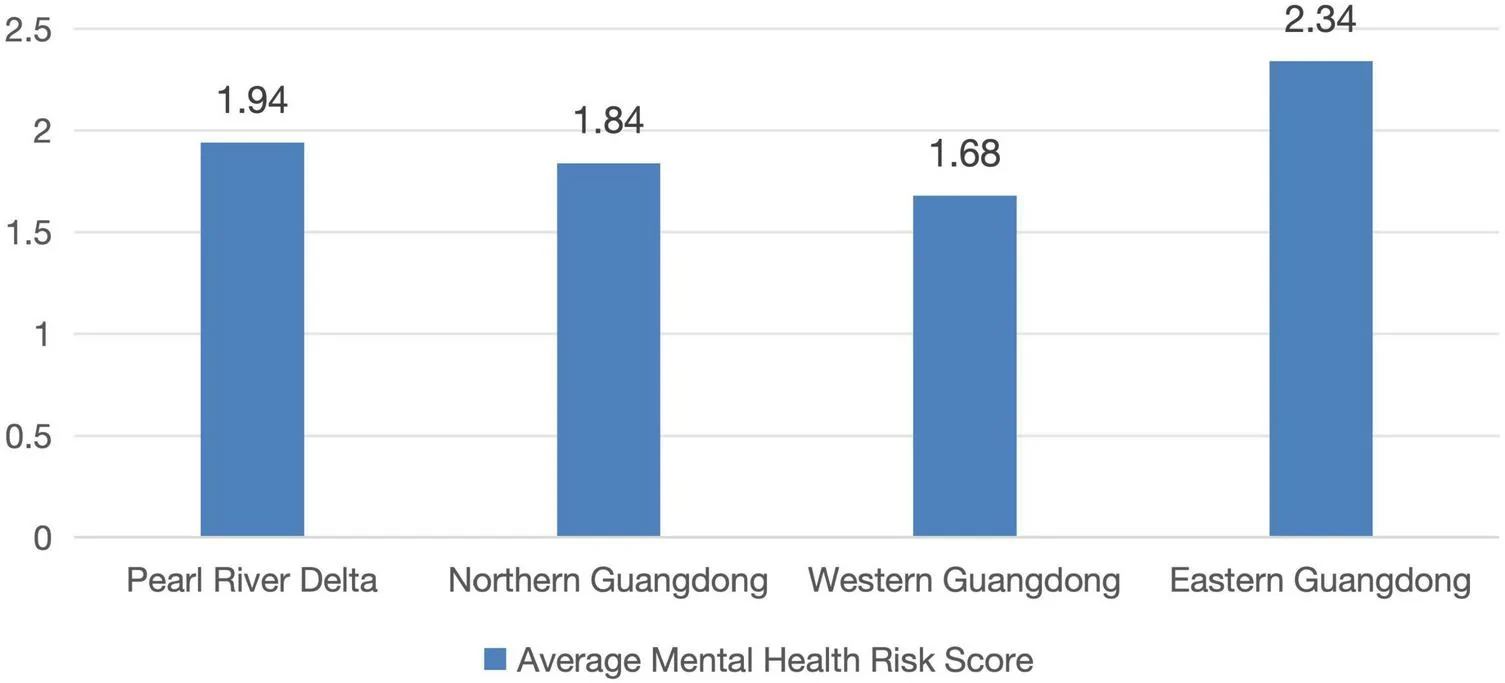
Differences in mental health risk scores across residential areas.

**FIGURE 10:**
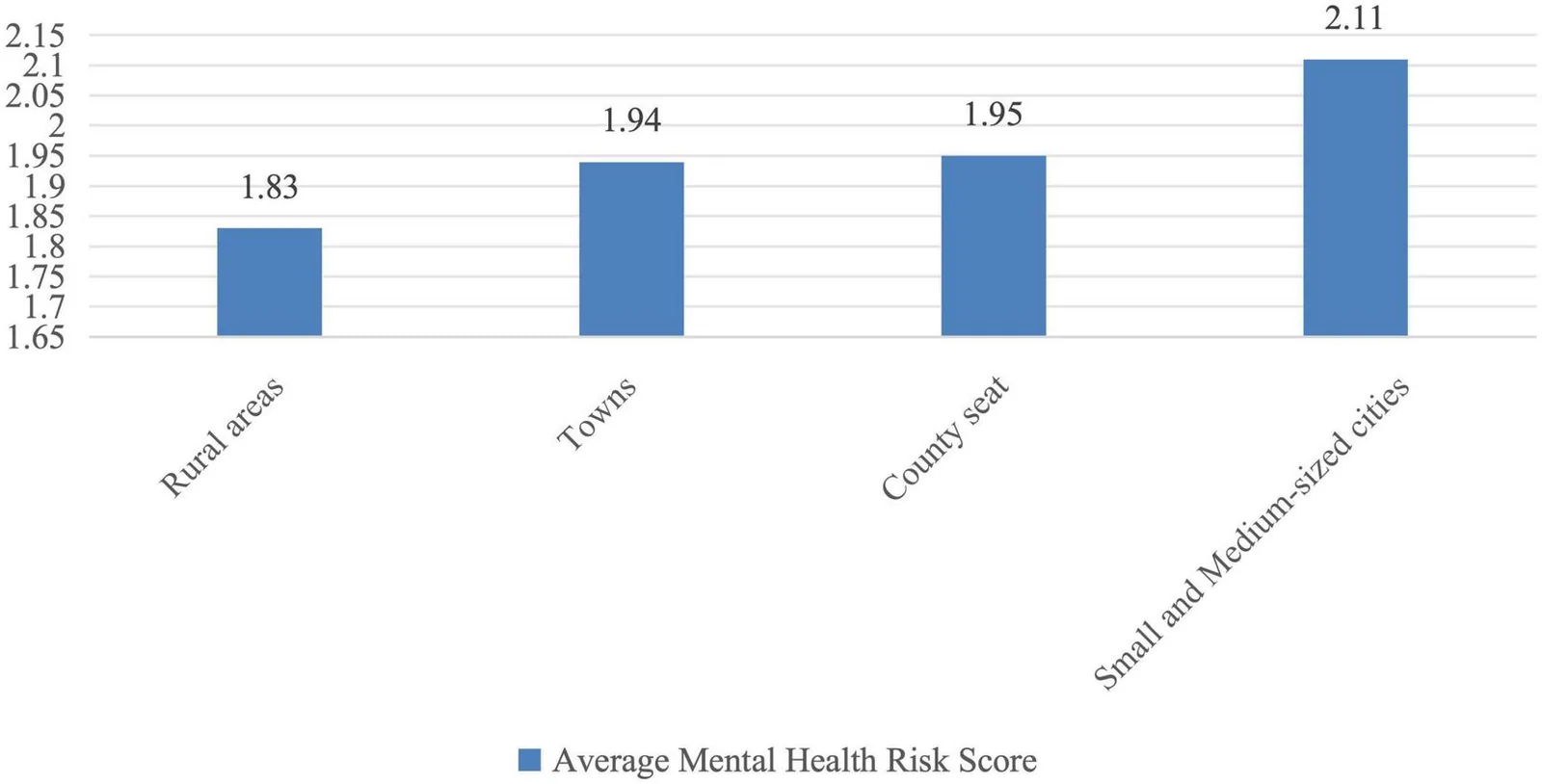
Differences in mental health risk scores across school districts.

**FIGURE 11:**
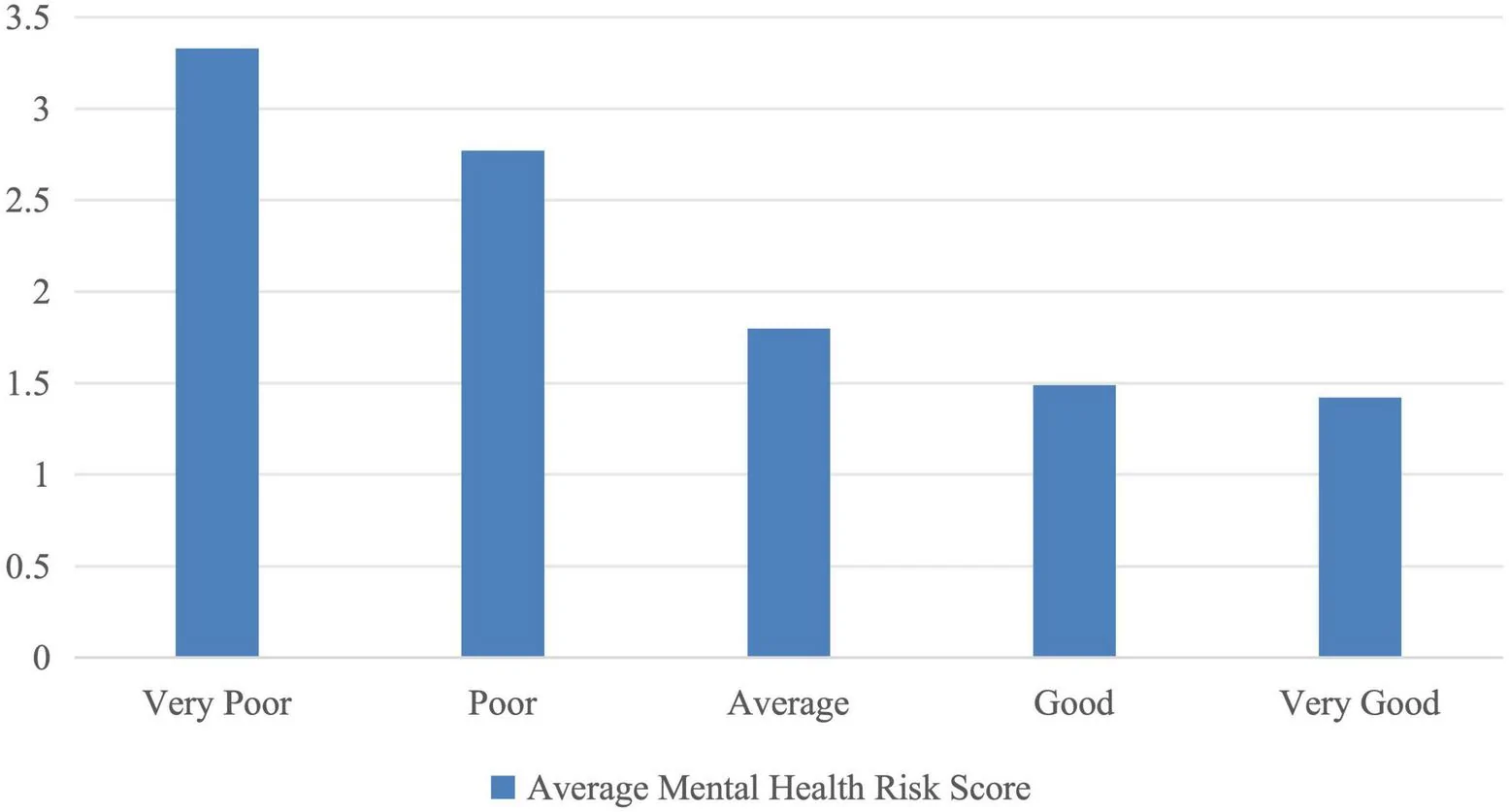
Differences in mental health risk scores by family economic status.

The survey indicates that among vocational secondary school students, 40.78% (12,646 persons) belong to the low-risk group, 39.46% (12,237 persons) to the moderate-risk group, and 19.76% (6,128 persons) to the high-risk group. Mental health risks are influenced by multiple factors, with the type of primary caregiver (Figure 8), region (Figure 9), place of residence (Figure 10), and family economic status (Figure 11) being the most significant. Groups with non-parents as primary caregivers exhibited relatively higher mental health risks; students in Eastern XX showed higher risks than those in other regions; those residing in small and medium-sized cities had relatively higher risks; simultaneously, perceived family socioeconomic status showed a significant negative correlation with mental health risks—that is, the lower the perceived socioeconomic status, the higher the mental health risk. It should be noted that the term “mental health risks” refers to psychological symptoms such as depressive symptoms, anxiety symptoms and sleep disorders. It must be clearly distinguished from “risks of psychological crisis behaviors” including self-harm, self-mutilation and suicide, so as to avoid misunderstandings of the overall situation caused by biased data interpretation.

### Lifestyle and physical/mental health status show significant covariation

#### Social media usage

**FIGURE 12:**
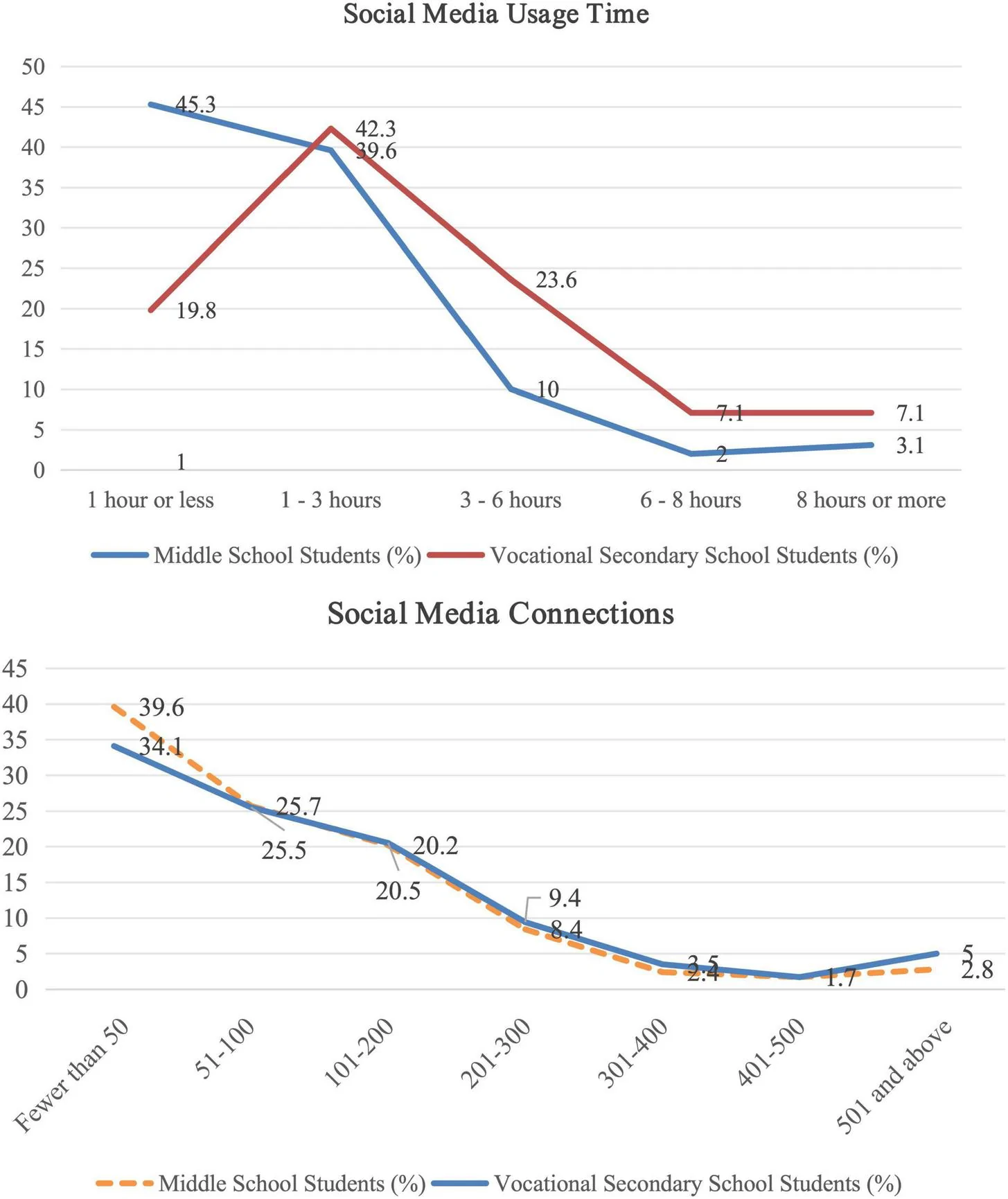
Social media usage time and number of platforms. Secondary school data is derived from a 2024 survey of over 800 junior and senior high school students at a XX secondary school.

Vocational students generally spent more time on social media and had more social media contacts than secondary school students (covering junior middle schools and general senior high schools) (Figure 12).

**FIGURE 13:**
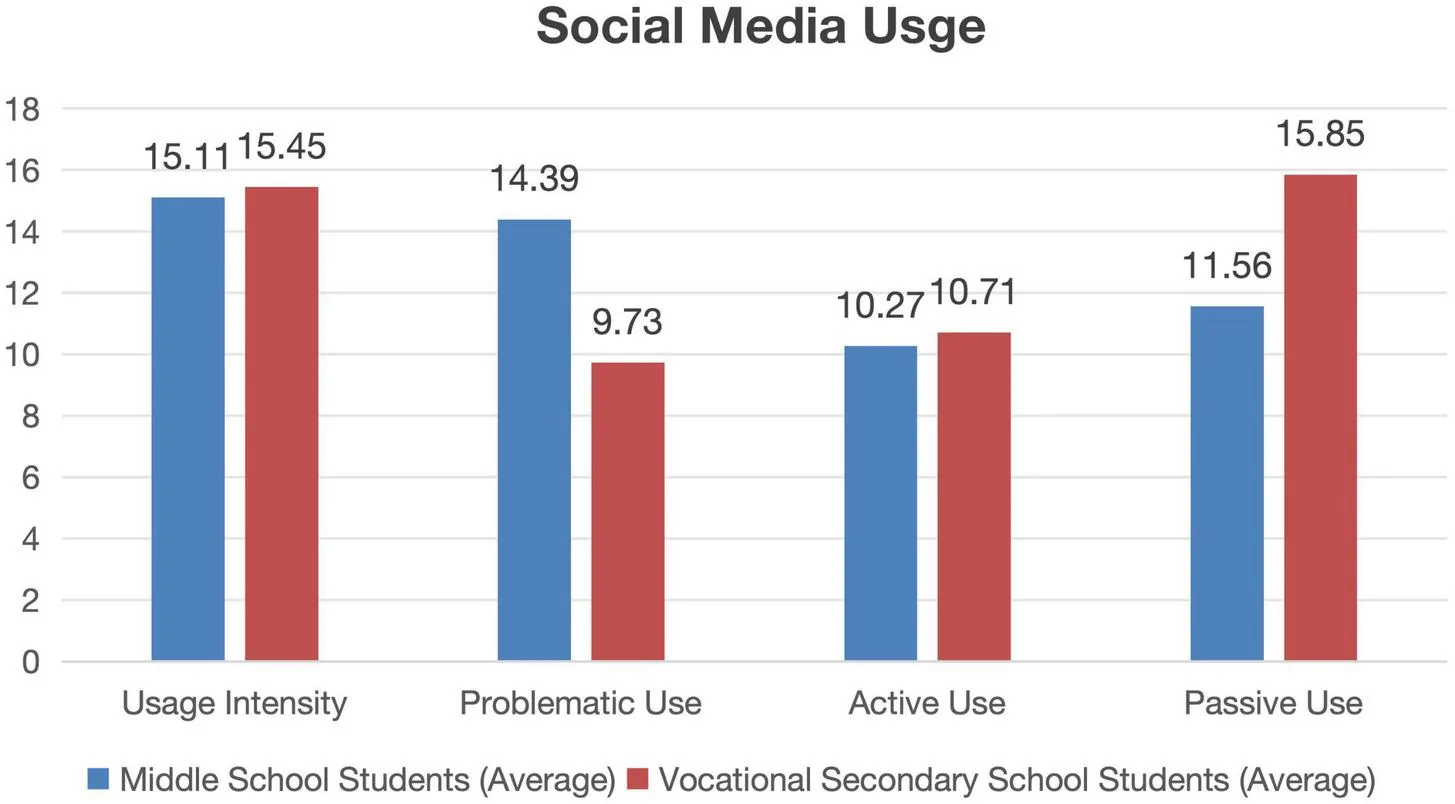
Comparison of social media usage scores between vocational students and secondary school students. Secondary school student data is derived from a 2024 survey of over 800 junior and senior high school students at a XX secondary school.

Vocational college students obtained higher scores in problematic use and passive use compared with middle school students, suggesting a greater tendency toward irrational screen-scrolling and passive information intake, as well as excessive social media dependence and poor self-management (Figure 13).

#### Physical exercise habits

**FIGURE 14:**
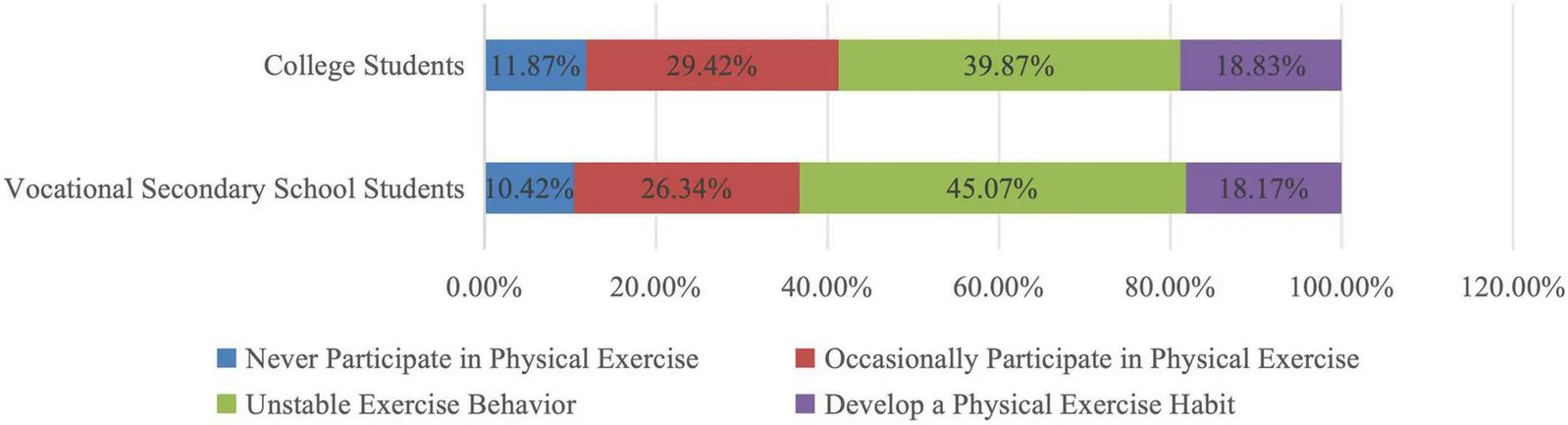
Physical exercise habits.

**FIGURE 15:**
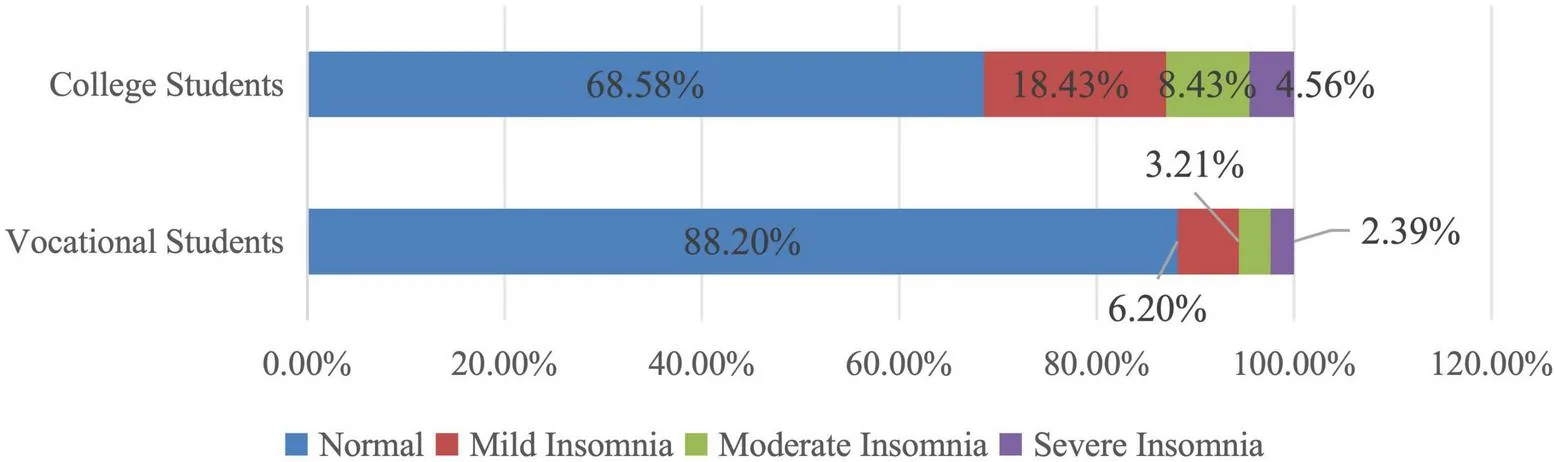
Insomnia among vocational students.

Vocational students generally exhibit better physical exercise habits than university students (Figures 14, 15), with over half maintaining regular exercise routines. Approximately 60% have established consistent exercise habits. However, 45.07% remain in an unstable exercise habit zone. This group may be disrupted by external factors such as academic pressure or seasonal changes, leading to reduced exercise frequency and consequent declines in physical fitness and psychological resilience.

#### Insufficient sleep

Overall, most students enjoy good sleep quality, outperforming university students in this regard (Figure 15). Nevertheless, insomnia remains prevalent, with severe cases requiring particular attention. Correlated with mental health data, insomnia may stem from psychological stressors. Recommendations include promoting healthy routines and implementing stress management strategies to enhance student wellbeing.

### Incidence of unhealthy habits and depression/mental health risks

**FIGURE 16:**
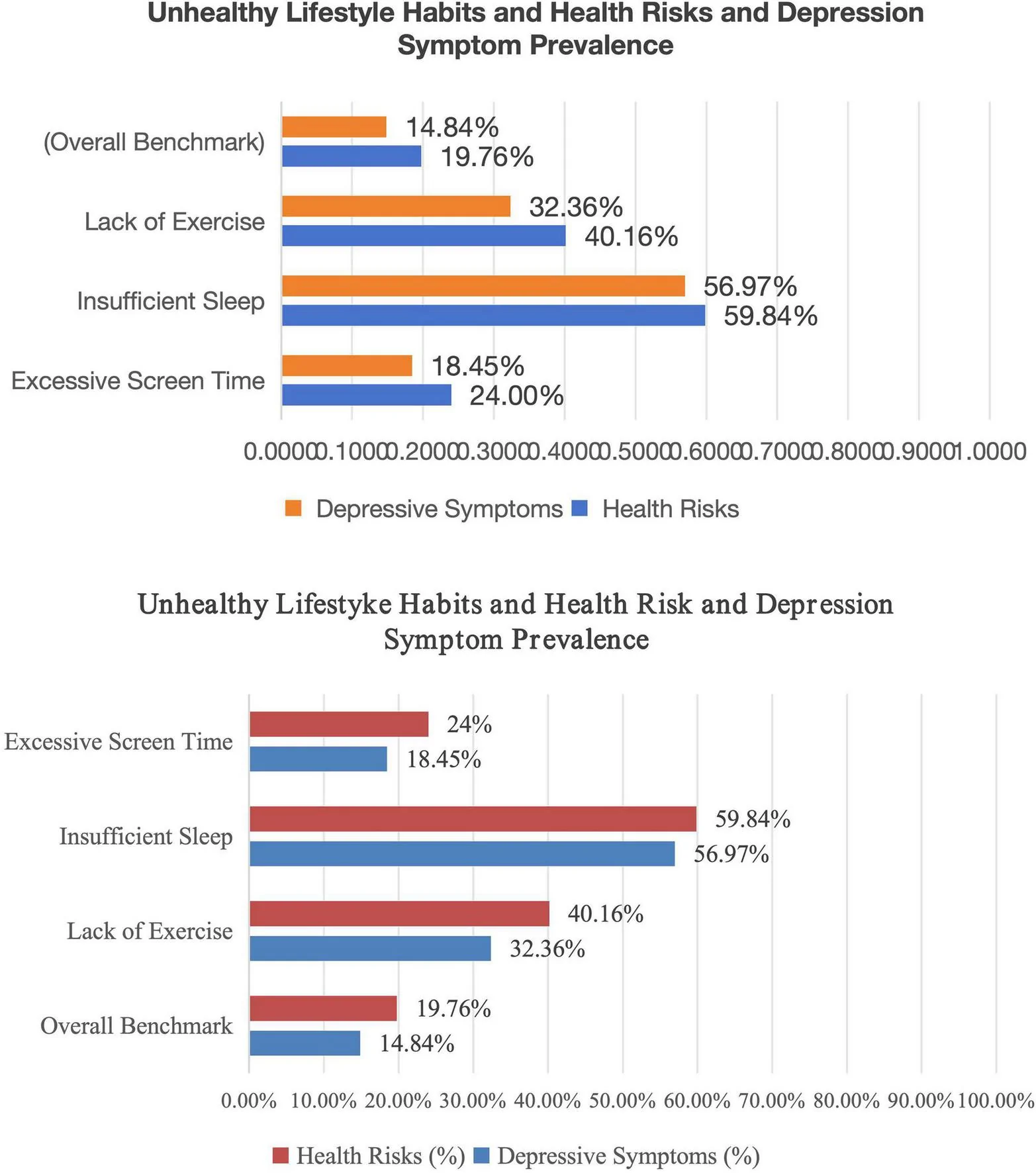
Incidence of unhealthy habits and mental health risks.

Data show that the prevalence of depressive symptoms is 14.84%, which serves as the baseline proportion of depressive symptoms for the entire sample; the prevalence of mental health risks stands at 19.00% (Figure 16).

Although the overall detection rate of depressive symptoms in the entire sample was 26.35%, specific baseline analyses regarding the impact of lifestyle factors revealed that students with healthy lifestyle habits consistently exhibited significantly lower baseline risk levels; conversely, the presence of unhealthy habits exacerbated these risks. Further analyses indicated that compared with students maintaining healthy habits, those lacking physical exercise, experiencing sleep deprivation, or excessively using electronic media demonstrated higher incidences of depressive symptoms and mental health risks. Overall, sleep and physical exercise serve as the core physiological recovery factors, and interventions for electronic device use should be evaluated in light of specific scenarios.

### On the prominence of academic burnout and insufficient achievement

**FIGURE 17:**
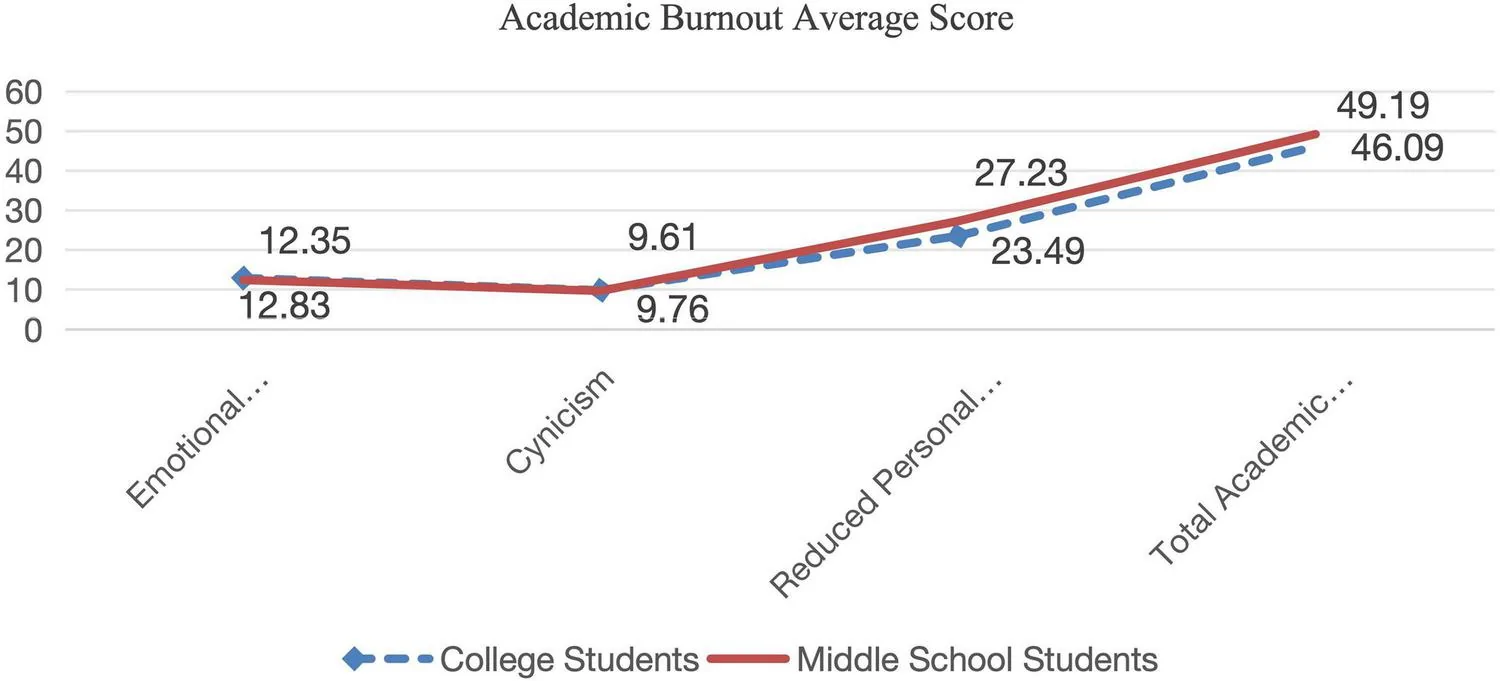
Comparison of academic burnout between vocational and college students.

The average academic burnout score among vocational students reached 49.19, slightly higher than the university student average, primarily characterized by low achievement satisfaction (Figure 17). This indicates that vocational students struggle to derive sufficient value from their current academic accomplishments. Prolonged exposure to low achievement satisfaction poses risks to students’ mental health and may lead to learned helplessness.

### Interpersonal relationships support quality requires enhancement

#### Peer relationships

**FIGURE 18:**
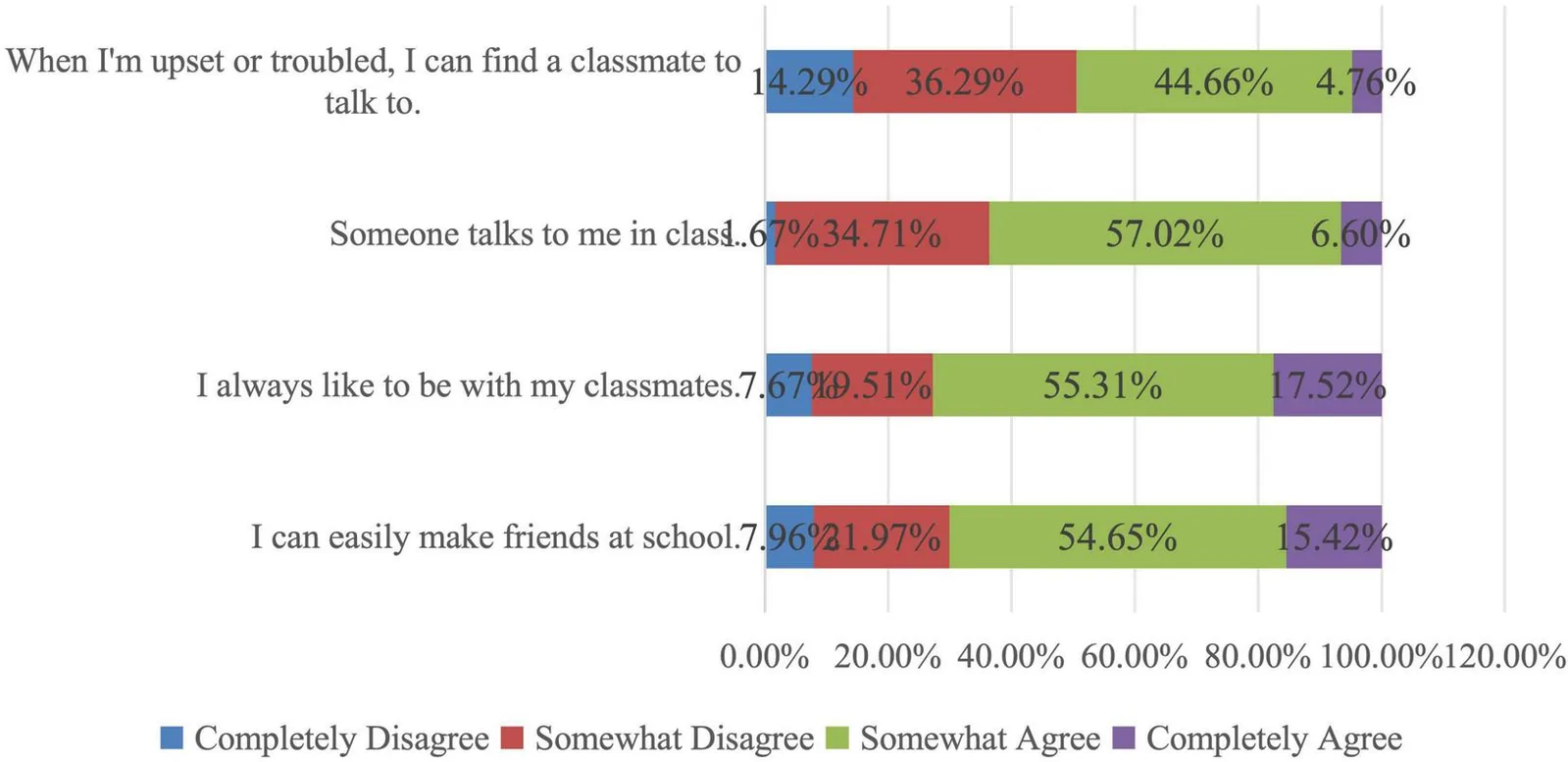
Peer relationships.

The peer relationship results indicate that students generally fare well (Figure 18). Across all four items, over half of the students demonstrated the willingness and ability to actively integrate into the group, suggesting a stable foundation for peer interaction in campus life. Meanwhile, it is noteworthy that more than 20% of students reported difficulty in making friends at school. They also lack reliable confidants to turn to when experiencing emotional distress and have insufficient channels to relieve psychological stress.

**FIGURE 19:**
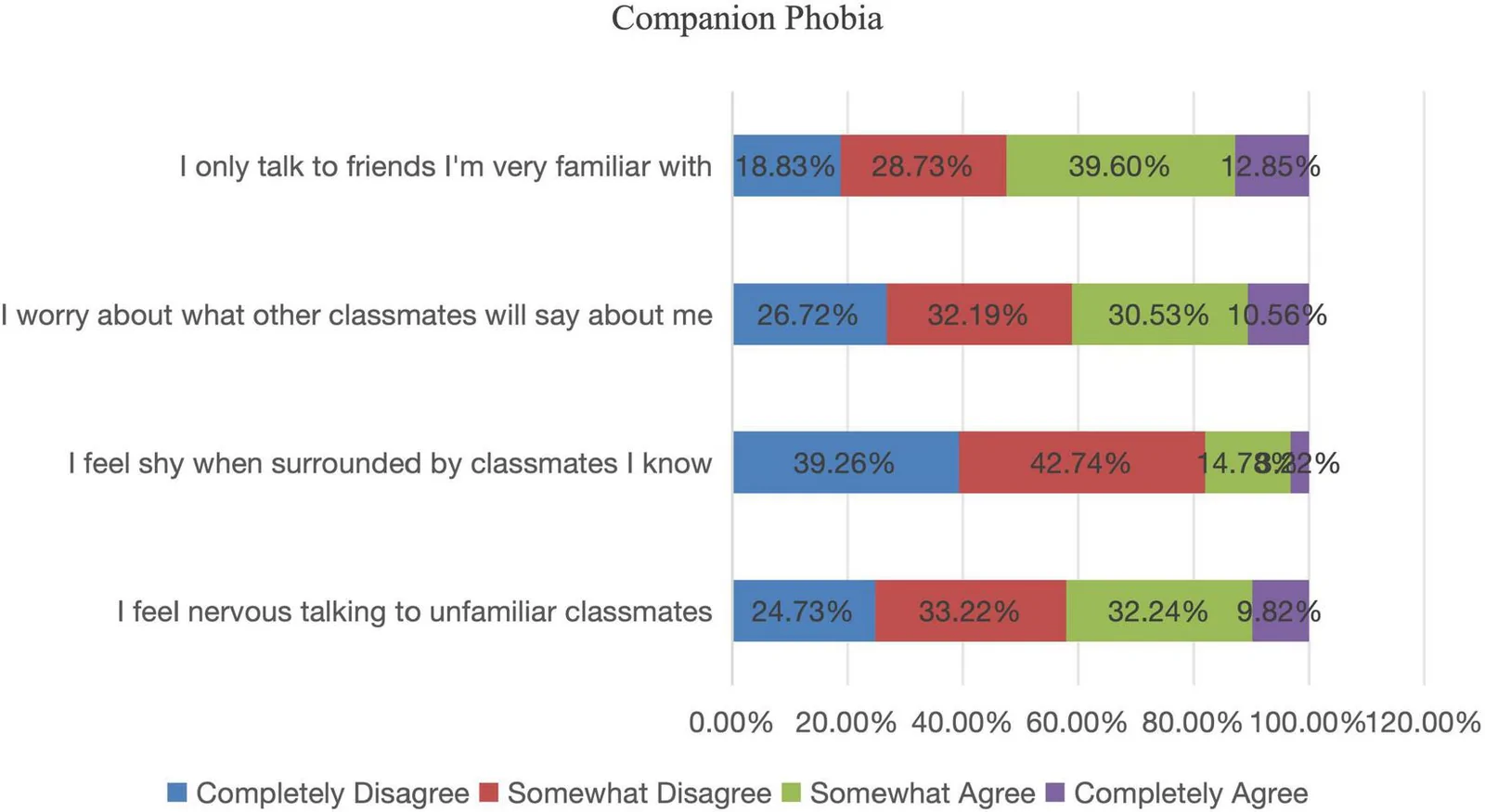
Peer fear.

Survey data indicates that students’ “peer fear” is characterized by a lack of social skills for interacting with strangers and concerns about others’ evaluations (Figure 19). Tension in unfamiliar situations is the most prominent issue, and evaluation sensitivity is widespread. This kind of anxiety can trigger social withdrawal. Over half of students communicate merely with familiar friends and depend on small-scale secure relationships.

#### Teacher-student relationships

**FIGURE 20:**
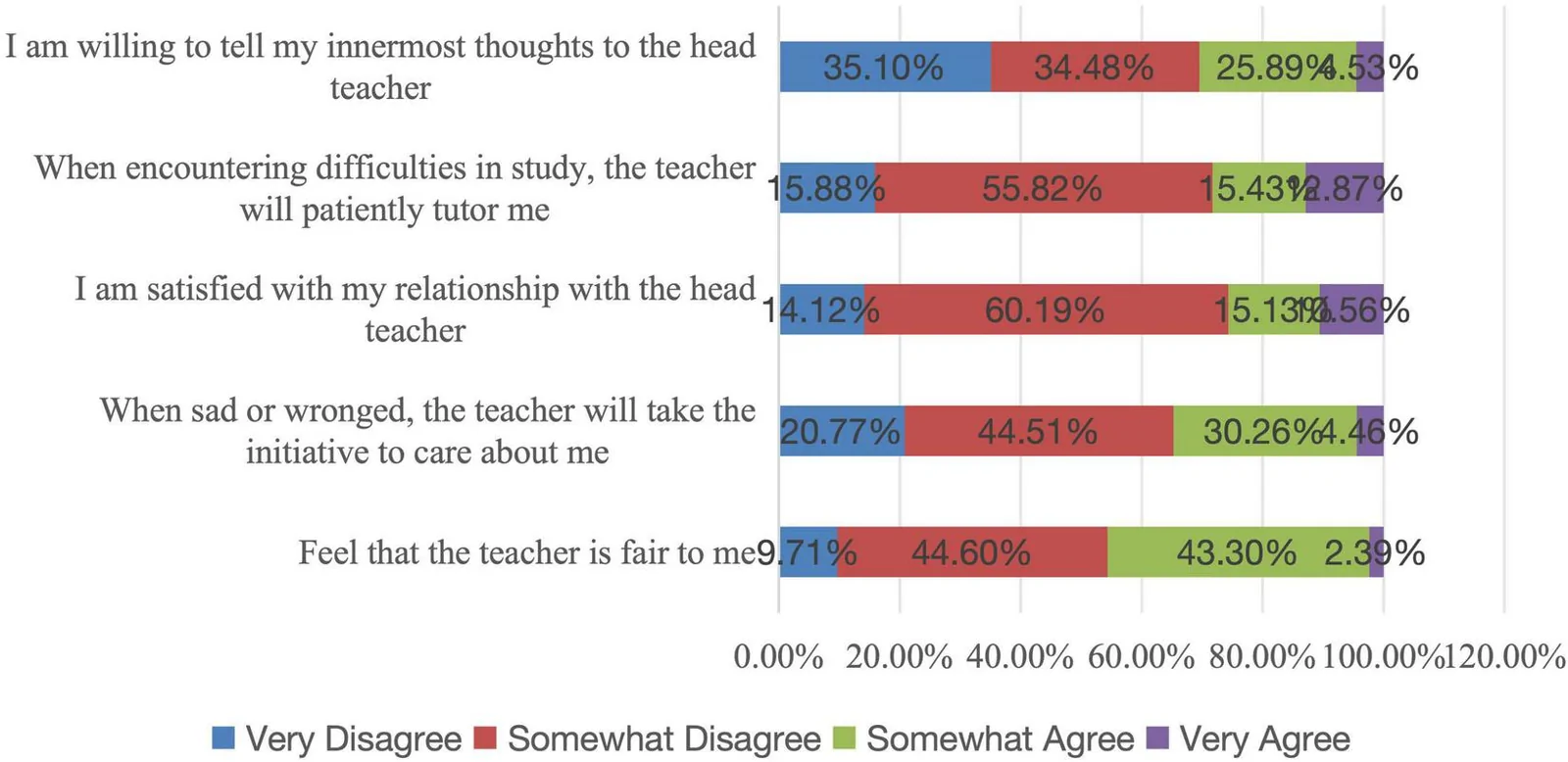
Teacher-student relationships.

Students’ trust and interaction with teachers exhibit a contradictory state of “high expectations and low initiative.” “High expectations” reflect an internal psychological tendency and state, while “low initiative” is a common characteristic among vocational students at this age, indicating their need to maintain a certain “safe and comfortable distance” from adults (Figure 20). This factor also indirectly corroborates the finding of relatively limited in-depth communication between teachers and students, with only 30.42% of students “willing to proactively seek teachers to share their troubles.”

#### Family function

**FIGURE 21:**
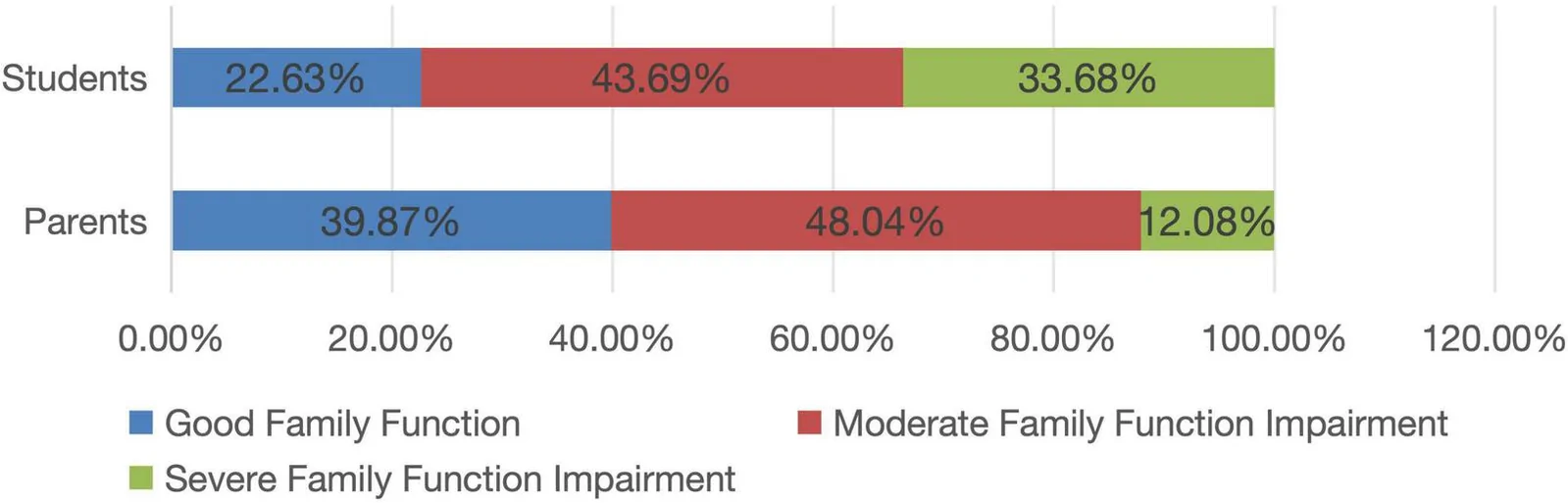
Comparison of family functioning.

A 33.68% of students perceive severe family dysfunction, while 43.69% report moderate dysfunction—combined, 77.37% indicate abnormal family functioning (Figure 21). Dysfunctional families limit students’ access to resources for emotional expression, secure attachment, and value reinforcement. This leaves them more vulnerable to isolation and emotional exhaustion when facing academic, interpersonal, and self-development pressures, resulting in heightened psychological fragility.

#### Offline interview research analysis

This offline study mainly adopted interviews as the research method, involving 671 secondary vocational school students and 197 class teachers. It mainly explores students’ concerns, the causes of psychological developmental barriers, and supportive approaches to cope with psychological distress. The key findings are presented as follows (Table 5):

**TABLE 5:** Statistical data on students’ psychological development concerns.

Category	Key terms	Percentage
Interpersonal relationships	Peer conflicts, parent-child conflicts, social difficulties	30.61%
Academic pressure	Grade anxiety, uncertainty about further education	25.51%
Mobile entertainment	Game dependency, short video addiction, virtual social dependency	20.41%
Self-identity	Appearance anxiety, sense of self-worth	15.31%
Family issues	Financial pressure, family conflicts	8.16%

#### Student psychological development concerns

The data (Table 5) indicates that a significant number of students tend to derive a sense of existence, adaptation, identity, and security from external conditions.

#### Sources of student psychological developmental barriers

Data shown that (Table 6), academic factors are the primary source of psychological distress, with learning ability anxiety and entrance exam pressure accounting for a combined 41.02%. This indicates that “academic performance and college admission prospects” are the most significant psychological stressors for current vocational students. Following this are interpersonal relationship concerns, including conflicts with classmates, teacher-student relationships, social anxiety, and appearance anxiety, collectively accounting for 23.90%. This reveals that students in the stage of exploring identity and group belonging are highly sensitive to others’ evaluations. Management-related issues and conflicts over autonomy (mobile phone restrictions, dissatisfaction with campus management) combined account for 14.08%, reflecting students’ prominent need for personal space and a sense of self-determination.

**TABLE 6:** Statistical data on sources of psychological developmental obstacles among students.

Type	Key terms	Quantity	Percentage
Learning ability anxiety	Subject imbalance, difficulty following lectures, weak foundational knowledge, poor study methods	287	22.21%
Pressure from entrance exams	Vocational college entrance exams, certification tests, internship evaluations, academic ranking	243	18.81%
Peer conflicts	Dormitory disputes, clique exclusion, verbal mockery	189	14.63%
Daily academic burden	Too much homework, frequent exams, long evening study sessions	121	9.37%
Conflicts over phone management	Confiscation of phones, gaming bans, communication restrictions	95	7.35%
Campus management dissatisfaction	Morning exercise system, cafeteria quality, dormitory conditions	87	6.73%
Family conflicts	Parental arguments, financial control, unrealistic expectations	76	5.88%
Social anxiety	Avoiding initiating conversations, feeling tense in public settings, fear of judgment	63	4.88%
Teacher-student relationships	Teachers showing favoritism, poor communication, criticism hurting self-esteem	58	4.49%
Body image anxiety	Obesity, acne, worsening myopia, developmental issues	42	3.25%
Financial pressure	Insufficient allowance, scams in part-time jobs, rising prices	31	2.40%

#### Support mechanisms for coping with psychological distress

Students prefer immediate, accessible, and everyday coping methods like emotional support and distraction—e.g., listening, eating treats, or hanging out with peers. Compared to parents and teachers, they are more likely to seek help from peers and use simple, unstructured approaches to regulate emotions (Table 7). While lacking professionalism, these strategies provide short-term relief from anxiety and unease, highlighting peer support’s inherent advantages in accessibility and trust.

**TABLE 7:** Statistical data on students’ coping support methods for psychological distress.

Type	Key terms	Quantity	Percentage	Typical examples
Emotional support	Listening, comforting, companionship	136	26.7%	Silently accompanying and comforting; serving as a confidant
Distraction	Eating delicious food, exercising, gaming, music	128	25.0%	Buy her treats; play games; listen to music
Solve problems	Offer advice, analyze problems, help resolve issues	87	17.0%	Help them analyze their current situation and how to resolve it; offer reasonable suggestions
Seek professional assistance	Seek help from teachers, psychologists, or external resources	65	12.7%	Take her to see a psychologist; seek assistance from teachers
Accompanying activities	Take walks, go out for fun, accompany her in doing something	61	11.9%	Play ball with him; take her out to relax
Self-regulation	Sleep, solitude, processing alone	27	5.3%	Sleep; listen to music to ease the mood
Class/environmental intervention	Class activities, school counseling, environmental improvements	7	1.4%	Provide students with an outlet for emotional release; organize more engaging activities for students

#### Supplementary explanation

This report compares the identified mental health indicators among vocational high school students with existing survey data from university students, regular junior and senior high school students, the general population nationwide, and young adults in Guangdong Province. These comparisons are intended solely as a reference for current status assessment and should not be used as a basis for rigorous statistical inference of intergroup differences, given that the two sample groups exhibit significant objective disparities across multiple dimensions and lack full comparability. The specific methodological differences are as follows:

(1) Different age ranges:

The study primarily targeted adolescents aged 16–18 attending secondary vocational schools; the junior and senior high school samples covered ages 12–17, while university students were predominantly aged 18–22. The national and Guangdong provincial youth samples spanned all age groups, demonstrating distinct differences in adolescent neurodevelopmental and academic growth stages across populations. Additionally, emotional regulation and stress sources exhibited inherent age-related stratification patterns.

(2) Significant differences exist in educational backgrounds and living environments:

Secondary vocational education focuses primarily on cultivating vocational skills and preparing students for employment, yet students generally face dual pressures of academic bias and career uncertainty; regular high schools prioritize exam preparation as the main source of stress; college students enjoy independent living conditions and diverse developmental options; whereas ordinary young adults and residents lack a unified educational environment within schools. These four groups exhibit entirely distinct growth objectives and daily stress patterns, and there is no unified benchmark for understanding the mechanisms underlying psychological risks.

(3) The investigation tools and grading criteria are not fully standardized:

Previous literature, the Blue Book, the scale versions used in university surveys, risk threshold criteria, and indicator definitions are not entirely consistent. For instance, some student meta-analyses employed simplified psychological screening tools; university surveys utilized customized intra-university scales; the National Mental Health Blue Book established cutoff scores for the entire population, which differ from the scoring methodology and risk determination thresholds of the specialized screening scale designed for secondary vocational schools, resulting in inconsistencies in the statistical interpretation of indicators.

(4) Data collection periods are inconsistent:

This survey was conducted from December 2024 to April 2025; the comparative data were sourced from the 2022 Guangdong Youth Epidemiological Survey, the 2024 National Mental Health Blue Book, the 2025 Psychological Census of Undergraduate Institutions, and previous meta-analytic studies on middle school students. Changes in socio-environmental factors, educational policies, and adolescent media usage habits across years have led to temporal fluctuations in baseline psychological indicators.

(5) There are differences in the sample sampling structure:

This survey covered 54 secondary vocational schools across eastern, western, and northern Guangdong as well as the Pearl River Delta region, accounting for typical family characteristics of secondary vocational education settings such as urban-rural disparities, families with multiple children, and intergenerational caregiving arrangements. However, the comparison sample’s sampling methods, urban-rural distribution ratios, and family structure composition did not align with those of this study, resulting in inconsistent sample composition.

Given the multiple methodological differences outlined above, this comparison across various groups serves only as a general reference and does not allow for rigorous statistical testing of differences. Furthermore, there is a scarcity of systematic mental health survey data available domestically covering a large cohort of vocational school students across the province, and no highly matched contemporary control samples exist at present. If high rigor is required in the report, all cross-group comparison charts and textual descriptions may be omitted entirely.

### Recommendations

#### Recommendations for student psychological development

Based on this survey, to advance the psychological education system in secondary vocational schools from “problem response” to “development promotion,” systematic efforts should be made in four areas: enhancing mental health literacy, optimizing growth ecosystems, strengthening teacher capabilities, and establishing crisis prevention systems.

### Implementing mental health literacy enhancement initiatives

#### For students: strengthen agency and self-growth capacity

Mental health education should shift from a “teacher-centered lecture mode” to a “student-participatory activity mode.” Structured peer support programs have been proven as effective approaches to promoting adolescent wellbeing and psychological resources during health crises (Pavarini et al., 2024). Through methods such as “thematic mental health class meetings,” “peer support groups,” or “peer education” (Widnall et al., 2024), students can engage in discussions around real-life difficulties (e.g., peer conflicts, test anxiety) and collectively explore feasible strategies. Under the guidance of school mental health teachers and head teachers, student organizations are encouraged to participate in co-creating mental health education resources, thereby enhancing the relevance, attractiveness, and perceptibility of mental health education.

#### For teachers: transforming psychological knowledge into actionable skills

Teachers are critically involved in the delivery of school-based mental health promotion (SMHP) interventions in school (Dinnen et al., 2024). Teachers need to acquire technical skills such as goal construction, teaching method design, and activity development. They should internalize their existing mental health knowledge into daily supportive and communication competencies. It is recommended to continuously promote the ABC Certificate Training on mental health education for head teachers. Teachers also report needing additional mental health resources, support, and training (Gunawardena et al., 2024). Scenario-based practice, group supervision, and experience sharing should be provided simultaneously, enabling teachers to identify students’ emotional changes, listen effectively, respond to psychological needs, and master basic crisis early warning skills.

#### For families: building a home-school collaborative support network

Insufficient family emotional support is an important contextual factor contributing to psychological distress among secondary vocational students. A positive family atmosphere is crucial to the healthy development of students (Biglan et al., 2012). Nurturing and connected parenting defines family relationships, which in turn promotes self-identity, self-esteem, and self-efficacy among children and adolescents (Hunter et al., 2015). These include good parental mental health, strong family cohesion, and high levels of trust between children and parents (Biglan et al., 2012).

Schools can enhance parents’ understanding of the psychological development patterns during adolescence and improve parent-child communication through practical activities such as Smart Parent Classes, home-school psychological growth communities, and family communication assignments, thereby strengthening students’ self-regulation abilities (Bachman et al., 2024). Parents with effective parenting experience should be encouraged to share their insights or engage in dialogues with mental health professionals to improve parental participation and practicality.

Meanwhile, schools can integrate mental health knowledge into daily educational scenarios by promoting mini psychological lectures, reading sessions, and situational dramas through campus official WeChat accounts, fostering a psychological culture of care, understanding, and empathy.

### Optimizing the student psychological growth ecosystem

#### Fostering exercise habits to enhance physical and mental resilience

Physical activity is closely associated with mental health, including such indicators as depression, stress, and life satisfaction (Rodriguez-Ayllon et al., 2019). Daily exercise can be incorporated into the conduct and class evaluation systems through a “fitness check-in points system” to boost participation intention. Based on physical fitness test results, personalized “exercise prescriptions” can be developed to enhance stress resilience and emotional stability through regular physical activity.

#### Establish a student psychological growth support platform

School-based program platform construction contributes positively to students’ mental health development (Shoshani and Steinmetz, 2014). For example, through initiatives like “Sunshine Angel” story sharing and “Happiness Bank” positive experience logs, guide students to express emotions and accumulate psychological resilience. Create traceable growth archives to shift psychological support from “problem-solving” to “daily nurturing.”

#### Leveraging peer support’s accessibility and trust

Peer support has a positive impact on mental health (White et al., 2020). Students with positive influence can be selected as “Sunshine Seeds” to form peer psychological growth groups (Repper and Carter, 2011). After receiving training in active listening, companionship, and observation skills, they serve as frontline touchpoints of the campus mental health support network, lowering the threshold for help-seeking and enhancing group belonging and security.

#### Promoting experiential and participatory growth curricula

Utilize art therapy, outdoor, and themed workshops to encourage students to express themselves, regulate emotions, and strengthen their sense of purpose and self-efficacy through co-creation and interaction. Integrate these with labor education and research-based learning courses to embed psychological growth into daily studies and campus culture development.

#### Organize “career exploration” and “interest matching” programs

Arrange “one-day immersion” experiences for students at vocational colleges and corporate workplaces. Combine these with Holland Career Interest Assessments to provide personalized pathway recommendations, helping students develop tangible future aspirations and alleviate feelings of confusion and low motivation.

### Empowering teachers’ psychological education capabilities

#### Enhance teacher support capabilities through practice-oriented training

It is essential for teachers to participate in school mental health services to improve their professional skills (Franklin et al., 2012). Through thematic workshops, case analyses, and scenario-based drills, teachers can enhance their capacities in empathetic communication, risk signal identification, classroom climate building, and basic psychological support, enabling them to become “educators who truly see and understand students.”

#### Establishing diverse collaborative growth mechanisms

Introducing municipal and provincial-level teams of distinguished homeroom teachers to establish a closed-loop support system encompassing “training—job shadowing—professional development—supervision.” This initiative prioritizes support for mid-career and young homeroom teachers, facilitating skill transfer and experience sharing to enhance the school’s overall capacity for psychological education.

### Establish a proactive psychological crisis prevention network

#### Dynamic monitoring and record-keeping

The use of standardized digital scales, the establishment of regular screening and tracking mechanisms, and the construction of digital psychological growth archives can effectively detect the mental health risks of non-clinical, school-going adolescents (Kadirvelu et al., 2026). Combined with daily observations by peer psychological committee members, homeroom teachers, and school psychologists, a dual-dimensional monitoring system of “quantitative + contextual” is formed.

#### Multi-role collaborative early warning system

Establish a collaborative mechanism linking peers, homeroom teachers, psychological counselors, parents, and professional institutions. Implement an early identification and tiered intervention model featuring daily observations, weekly feedback, and monthly consultations.

#### Transition from vigilant prevention to supportive prevention

Implement the “Listening Companion Program” to enhance warmth in teacher-student relationships and advance the “universal mentoring system” to establish long-term companionship, providing students with stable, reliable, and sustainable psychological safety support.

## Discussion

This study provides a comprehensive analysis of the mental health status of secondary vocational school students based on a large-scale sample of over 31,000 participants. The findings reveal that although the overall mental health status appears relatively stable, several critical issues persist, including low levels of mental health literacy, high prevalence of depressive symptoms, significant academic burnout, and insufficient family and social support. These results highlight the coexistence of “surface stability” and “structural vulnerability” within this population.

First, the prevalence of depressive symptoms (26.35%) and suicidal ideation (20.35%) indicates that mental health risks among secondary vocational students remain at a relatively high level. Compared with general secondary school populations, vocational students appear to face additional stressors related to academic pathways, career uncertainty, and social identity. These findings are consistent with prior research suggesting that adolescents in transitional educational tracks are more vulnerable to psychological distress due to heightened uncertainty and perceived limited upward mobility. Furthermore, the widespread presence of academic burnout, particularly low achievement satisfaction, suggests that students struggle to derive meaning and value from their academic experiences. Prolonged exposure to such conditions may contribute to learned helplessness and reduced motivation, thereby exacerbating mental health risks.

Second, existing research has confirmed that lifestyle factors affect mental health status. Students with insufficient physical exercise, poor sleep quality, and excessive social media use exhibited higher levels of depressive symptoms and psychological risks (Yu and Huang, 2023). This aligns with existing evidence that health-related behaviors are closely associated with adolescents’ emotional regulation and psychological wellbeing. Notably, although a proportion of students reported regular exercise habits, a considerable number remained in an unstable behavioral pattern, indicating that external environmental factors may disrupt healthy routines. These findings suggest that promoting stable and structured daily habits is essential for strengthening psychological resilience.

Third, social support systems—particularly family and school relationships—play a crucial role in shaping students’ mental health. A substantial proportion of students reported moderate to severe family dysfunction, which limits access to emotional support, secure attachment, and value reinforcement. As a result, these students are more likely to experience loneliness, emotional exhaustion, and psychological vulnerability when facing academic and interpersonal challenges. In addition, although students expressed high expectations toward teachers, their willingness to actively seek help remained low, reflecting a gap between perceived support and actual utilization. This “high expectation–low initiative” pattern underscores the need to improve the accessibility and responsiveness of school-based support systems.

Fourth, peer relationships emerge as a double-edged factor. While peer groups provide accessible emotional support and a sense of belonging, they may also contribute to social anxiety and peer-related stress, particularly in the form of fear of negative evaluation. The findings from both survey and interview data indicate that students tend to rely more on informal peer support than on formal psychological services. This highlights the importance of integrating peer-based support mechanisms into the school mental health system, while also providing appropriate guidance to ensure their effectiveness and safety.

Based on these findings, this study suggests that psychological education in secondary vocational schools should shift from a problem-oriented approach to a developmental and preventive framework. Rather than focusing solely on crisis intervention, schools should prioritize the construction of a comprehensive psychological growth ecosystem. This includes enhancing students’ mental health literacy and self-regulation capacities, strengthening teachers’ practical competencies in psychological support, promoting family–school collaboration, and establishing multi-level early warning and intervention systems. Such an integrated approach can improve both the accessibility and effectiveness of mental health services and support the long-term development of students.

Despite its strengths, including a large sample size and a mixed-methods design, this study has several limitations. First, the cross-sectional nature of the data limits causal inference between variables. Second, the reliance on self-reported measures may introduce reporting bias. Third, the study focuses on a single province, which may limit the generalizability of the findings to other regions. Future research could adopt longitudinal designs, incorporate multi-informant data, and expand the geographical scope to further validate and extend the current findings.

In conclusion, this study highlights the urgent need to strengthen psychological education systems for secondary vocational students. Addressing mental health challenges requires coordinated efforts across students, teachers, families, and schools. Building a supportive, inclusive, and development-oriented psychological ecosystem is essential for promoting sustainable mental health outcomes in this population.

## Conclusion

### Empirical findings

Based on a large-scale survey of secondary vocational school students, this study identified several salient mental health patterns. First, a considerable proportion of students exhibited elevated levels of depressive symptoms and psychological distress, indicating a high-risk mental health profile within this population. Second, academic burnout emerged as a prominent issue, characterized by emotional exhaustion and reduced learning engagement. Third, deficiencies in family support and broader social support systems were significantly associated with poorer mental health outcomes, suggesting that external environmental factors play a critical role in shaping students’ psychological wellbeing.

### Practical implications

Based on these findings, several targeted strategies are recommended. At the school level, it is necessary to establish a tiered mental health education system that integrates universal prevention, early screening, and targeted intervention. Schools should strengthen the professional capacity of teachers and psychological counselors to identify and respond to students at risk.

At the family level, parent education programs should be implemented to enhance caregivers’ awareness of adolescent mental health and improve family support quality.

At the system level, greater coordination among schools, families, and community mental health services is required to build a collaborative support network. In particular, efforts should be made to shift from reactive, problem-oriented approaches toward proactive, development-oriented mental health promotion models.

## Data Availability

The original contributions presented in the study are included in the article/supplementary material, further inquiries can be directed to the corresponding author.
